# Catalysis of “outer-phase” oxygen atom exchange reactions by encapsulated “inner-phase” water in {V_15_Sb_6_}-type polyoxovanadates†
†Electronic supplementary information (ESI) available: IR spectra, thermogravimetric analysis data, powder diffraction patterns, crystal morphology data, details of solubility studies, additional crystallographic and magnetochemical data and comment on the unassigned signals in the ESI mass spectra. CCDC 1432847–1432850. For ESI and crystallographic data in CIF or other electronic format see DOI: 10.1039/c5sc04571a


**DOI:** 10.1039/c5sc04571a

**Published:** 2016-01-08

**Authors:** Michael Wendt, Ulrike Warzok, Christian Näther, Jan van Leusen, Paul Kögerler, Christoph A. Schalley, Wolfgang Bensch

**Affiliations:** a Institut für Anorganische Chemie , Christian-Albrechts-Universität zu Kiel , Max-Eyth-Str. 2 , 24118 Kiel , Germany . Email: wbensch@ac.uni-kiel.de; b Institut für Chemie und Biochemie der Freien Universität , Takustr. 3 , 14195 Berlin , Germany . Email: c.schalley@fu-berlin.de; c Institut für Anorganische Chemie , RWTH Aachen , Landoltweg 1 , 52074 Aachen , Germany

## Abstract

Antimonato polyoxovanadate (POV) cluster compounds {M(en)_3_}_3_[V_15_Sb_6_O_42_(H_2_O)_*x*_]·*n*H_2_O (M = Fe^II^, Co^II^, Ni^II^ and *x* = 0 or 1) obtained under solvothermal conditions exhibit unusual high water solubility making these compounds promising synthons for generation of new POV structure types. Electrospray ionization mass spectrometry provides evidence (i) for a water molecule encapsulated inside the cavity of a fraction of the spherical cluster shells, (ii) for a post-functionalization in water, namely a slow exchange of VO against Sb_2_O, (iii) for the inner-phase reactivity of the encapsulated water that is capable of opening an oxo-bridge, and (iv) for a significant acceleration of the ^16^O/^18^O exchange reactions of oxygen atoms in the cluster periphery with surrounding H_2_^18^O, when encapsulated water is present. To the best of our knowledge, this is the first example in polyoxovanadate chemistry for the transduction of inner-phase reactivity of an encapsulated guest molecule into changes in the outer-phase reactivity of the cluster. Magnetic susceptibility measurements reflect the individual contributions of the frustrated {V_15_} spin polytope and the {M(en)_3_}^2+^ complexes, with very weak coupling between these groups.

## Abstract 2

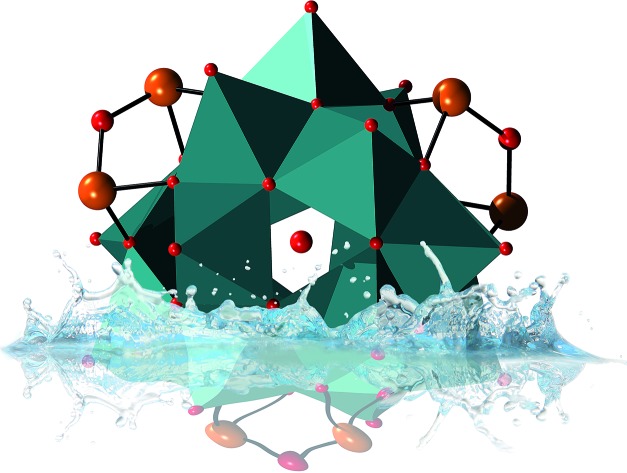
A water molecule encapsulated inside water-soluble {V_15_Sb_6_} antimonato polyoxovanadate cages accelerates oxygen-exchange reactions in the cluster periphery.

## Introduction

The chemistry of high-nuclearity polyoxomolybdate and polyoxotungstate (POMs) cluster shells significantly differs from that of polyoxovanadate clusters (POVs): (i) POMs are generally synthesised in acidic media, while POVs are usually prepared under basic conditions; (ii) POMs are mostly obtained applying soluble precursors consisting of pre-formed POM cluster shells, while POVs are synthesised from NH_4_VO_3_, V_2_O_5_, or VOSO_4_ because no soluble pre-formed hetero-POV clusters are at hand; (iii) POMs normally crystallise under ambient conditions, whereas POVs often require solvothermal reactions; (iv) in the overwhelming number of structures of POM clusters MO_6_ octahedra (M = Mo, W) are observed, while most POV cluster shells contain interconnected VO_5_ square pyramids; (v) in POMs the metal centres are generally in the highest oxidation state, whereas POVs are characterised by V^IV^ or mixed-valent V^IV^/V^V^ centres. Both groups of cluster compounds can be chemically and structurally modified by either attaching further building blocks such as transition metal complexes to the cluster shell or by replacement of Mo, W or V by heteroatoms.^1^

A unique class of heteroatom-modified POV clusters was discovered more than 25 years ago by Müller *et al.* who reported the first As-POV with the chemical formula [V^IV^_15_As_6_O_42_(H_2_O)]^6–^, which can be structurally derived from the {V_18_O_42_} archetype structure.^2^ Since then, several heteroatom-modified POV compounds containing As, Si, Ge and even Sb were discovered and characterised.^3^–^5^

Only little is known about the reactivity of polyoxovanadate clusters in solution despite of the increasing number of reports on novel POVs. This might be traced back to the fact that, in contrast to POMs, most POVs with high-nuclearity cluster shells are practically insoluble. This is a significant drawback compared to POM chemistry, because only a few V containing compounds are available that can be used as the starting material for the preparation of new POVs (*e.g.* {V_10_O_28_})^6^–^8^ but they do not include heteroatoms. In addition, nuclearity of the vanadate species in solution strongly depends on the pH value and polymerization at low pH resulting in formation of iso-POVs.^9^ If a well soluble hetero-POV were at hand, it could be applied as a precursor in post-functionalisation studies to prepare new polyoxovanadate clusters. This strategy is well established for POMs, but virtually unknown for POVs. In this context, some fundamental questions arise concerning the stability of such POV clusters in solution, for example whether they will be intact or transform into other clusters or fragments and how this will depend on encapsulated guest molecules. A few studies on reactions of encapsulated or templating guest ions in POM clusters have unravelled some intriguing reactivity.^10^ Analogous cases are unknown so far for POV clusters.

In this context, electrospray ionisation mass spectrometry (ESI-MS) has become a valuable tool in oxo-cluster chemistry and a significant body of knowledge has been acquired on the MS investigation of polyoxometallates.^11^ ESI-MS experiments range from the clusters' analytical characterisation^12^ to studies of their solution reactivity^13^ and to gas-phase experiments aiming at unravelling reaction patterns in the gas phase^14^ including the activation of small molecules such as methane.^15^ Mass spectrometry together with isotope exchange reactions can provide profound insight into cluster reactivity as evidenced by elegant studies of Schüth *et al.* on silicate clusters.^16^ ESI-MS experiments depend on the availablility of soluble samples. Consequently, detailed studies into the reactivity of POVs in solution by mass spectrometry are virtually unknown.

Here, we report the synthesis of three new compounds **I–III** with the composition {M(en)_3_}_3_[V_15_Sb_6_O_42_(H_2_O)_*x*_]·*n*H_2_O (*x* = 0, 1; *n* ≈ 15; M = Ni^II^ (**I**), Co^II^ (**II**), Fe^II^ (**III**); en = ethylenediamine), that all crystallise in the non-centrosymmetric monoclinic space group *C*2. A second pseudopolymorph (**IV**), {Ni(en)_3_}_3_[V_15_Sb_6_O_42_ (H_2_O)_*x*_]·*n*H_2_O (*n* ≈ 28) crystallising in the trigonal space group *P*321 has been obtained by altering the synthesis conditions. In the present contribution, we report solvothermal syntheses, crystal structures, magnetic properties and electrospray ionisation mass spectrometric studies of cluster reactivity in water. Interestingly, these POVs exhibit a strikingly good solubility in water. Thorough ESI-TOF MS studies on **I** provide evidence for the occurrence of intact clusters in solution and the time-dependent transition of the {V_15_Sb_6_} cluster shell into the Sb-richer {V_14_Sb_8_} cluster at room temperature. Furthermore, ^16^O/^18^O exchange studies demonstrate that the rate of oxygen exchange is significantly higher, when a single water molecule is encapsulated within the cluster's cavity. This “inner-phase” water molecule thus affects strongly the “outer-phase” reactivity of the cluster with water and catalyses the oxygen exchange in the clusters' peripheries.

## Experimental

### General

CHN elemental analysis was done with a EUROEA Elemental Analyzer (EURO VECTOR Instruments and Software). IR spectroscopy (400–4000 cm^–1^) was performed at room temperature using a Genesis FTIRTM spectrometer (ATI Mattson). Differential thermal analysis and thermogravimetry (DTA–TG) were carried out in nitrogen atmosphere (purity: 5.0; heating rate 1 K min^–1^; flow rate: 75 mL min^–1^; Al_2_O_3_ crucibles) using a Netzsch STA-409CD instrument. Energy dispersive X-ray analyses (EDX) and scanning electron microscopy (SEM) investigations were performed with a Philips Environmental Scanning Electron Microscope ESEM XL30 equipped with an EDX detector. X-Ray powder patterns were recorded on a STOE STADI-P diffractometer in transmission geometry (Cu-Kα_1_ radiation, *λ* = 1.540598 Å; Ge monochromator; flat sample holders). The phase purity of the reaction products becomes obvious when the experimental patterns are compared with those calculated from single-crystal X-ray data. UV/Vis spectra were recorded on an Agilent 8453 spectrophotometer from Agilent Technologies, Waldbronn, in a wavelength range from 190 nm–1100 nm (deviation: ±0.5 nm, wavelength reproducibility: ±0.02 nm).

### Syntheses

All chemicals (NH_4_VO_3_, Sb_2_O_3_, NiCl_2_·6H_2_O (Merck); CoCl_2_·6H_2_O, FeCl_2_·4H_2_O (Fluka); ethylenediamine (Grüssing); 1-(2-aminoethyl)piperazine (Alfa Aesar)) were purchased and used without further purification. All compounds were prepared under solvothermal conditions in DURAN® glass tubes (inner volume 11 mL) at 150 °C for 7 d using similar ratios for the reactants (see below for exact amounts used). After cooling to room temperature, the products were filtered off, washed with water and ethanol and dried *in vacuo*. The compounds were obtained as brown crystals. Compounds **I–III** could be prepared within a wide temperature range from 120–160 °C and the first crystals were observed after 3 d reaction time. Remarkably, crystals of **IV** were observed, when 1-(2-aminoethyl)piperazine was added to the reaction slurry and the reactant ratios were slightly altered compared to those for **I–III**. The role of 1-(2-aminoethyl)piperazine for product formation is not clear. In the following, the reaction conditions giving the best yields are summarised. The Ni and Co containing compounds (**I** and **II**) crystallised also applying an en : H_2_O ratio of 1 : 3, while **III** could only be obtained for a fixed en:H_2_O ratio of 1 : 5.

#### {Ni(en)_3_}_3_[V_15_Sb_6_O_42_(H_2_O)_*x*_]·*n*H_2_O (*n* ≈ 15) in *C*2 (**I**)

A solution of 1.7 mL ethylenediamine (25.4 mmol) and 2.3 mL H_2_O was added to a mixture of 0.1573 g (1.34 mmol) NH_4_VO_3_, 0.3081 g (1.06 mmol) Sb_2_O_3_ and 0.1565 g (0.658 mmol) NiCl_2_·6H_2_O. The yield based on V was 86%. Elemental analysis: C 7.43, H 3.19, N 8.64%; calc. (C_18_H_90_N_18_Ni_3_V_15_Sb_6_O_51_): C 7.10, H 2.98, N 8.28%. EDX analysis: V 46.0%, Sb 44.3%, Ni 9.7%; calc. (V_15_Sb_6_Ni_3_): V 45.7%, Sb 43.7%, Ni 10.6%.

#### {Co(en)_3_}_3_[V_15_Sb_6_O_42_(H_2_O)_*x*_]·*n*H_2_O (*n* ≈ 15) (**II**)

A solution of 1.7 mL (25.4 mmol) ethylenediamine and 2.3 mL H_2_O was mixed with 0.1573 g (1.34 mmol) NH_4_VO_3_, 0.3095 g (1.06 mmol) Sb_2_O_3_ and 0.2443 g (1.03 mmol) CoCl_2_·6H_2_O. The yield based on V was 48%. Elemental analysis: C 7.14, H 3.03, N 8.43%; calc. (C_18_H_90_N_18_Co_3_V_15_Sb_6_O_51_): C 7.10, H 2.98, N 8.28%. EDX analysis: V 45.7%, Sb 43.9%, Co 10.4%; calc. (V_15_Sb_6_Co_3_): V 45.7%, Sb 43.7%, Co 10.6%.

#### {Fe(en)_3_}_3_[V_15_Sb_6_O_42_(H_2_O)_*x*_]·*n*H_2_O (*n* ≈ 15) (**III**)

2.3 mL (34.4 mmol) ethylenediamine and 1.7 mL H_2_O were added to a mixture of 0.1571 g (1.34 mmol) NH_4_VO_3_, 0.3095 g (1.06 mmol) Sb_2_O_3_ and 0.1313 g (0.660 mmol) FeCl_2_·4H_2_O. The yield based on V was 68%. Elemental analysis: C 7.65, H 2.92, N 8.53%; calc. (C_18_H_90_N_18_Fe_3_V_15_Sb_6_O_51_): C 7.12, H 2.99, N 8.30%. EDX analysis: V 46.5%, Sb 43.2%, Fe 10.3%; calc. (V_15_Sb_6_Fe_3_): V 46.0%, Sb 44.0%, Fe 10.0%.

#### {Ni(en)_3_}_3_[V_15_Sb_6_O_42_(H_2_O)_*x*_]·*n*H_2_O (*n* ≈ 28) in *P*321 (**IV**)

A solution of 3 mL (22.9 mmol) 1-(2-aminoethyl)piperazine, 1 mL H_2_O and 0.15 mL ethylenediamine (2.24 mmol) was added to a mixture of 0.1177 g (1.00 mmol) NH_4_VO_3_, 0.2326 g (0.800 mmol) Sb_2_O_3_ and 0.1260 g (0.531 mmol) NiCl_2_·6H_2_O. The yield based on V was 54%. Elemental analyses: C 7.58, H 2.61, N 8.73%; calc. (C_18_H_72_N_18_Ni_3_V_15_Sb_6_O_42_): C 7.50, H 2.52, N 8.74%. EDX analysis: V 45.6%, Sb 44.1%, Ni 10.3%; calc. (V_15_Sb_6_Ni_3_): V 45.7%, Sb 43.7%, Ni 10.6%.

### Single-crystal structure analysis

Data collection was performed with a STOE Imaging Plate Diffraction System (IPDS-1) with Mo-Kα radiation (*λ* = 0.71073 Å). The crystal structures were solved with the program SHELXS-97 (ref. 17) and refined against *F*^2^ using SHELXL-97 (ref. 18) for **II–III**, for **IV** with the version of 2013 and for **I** with the version of 2014. All non-hydrogen atoms were refined anisotropically. The C–H and N–H hydrogen atoms were positioned with idealised geometry and refined using a riding model. Water hydrogen atoms could not be located. A numerical absorption correction was performed (min./max. transmission: 0.4902/0.6693 for **I**, 0.5249/0.6407 for **II**, 0.3124/0.7136 for **III** and 0.5900/0.6543 for **IV**). The absolute structures were determined and agree with the selected setting (Flack *x*-parameter: –0.02(2) for **I**, –0.07(3) for **II**, –0.006(19) for **III** and –0.028(19) for **IV**). In total, nine water molecules could be located during structure refinements. After structure refinement of compounds **I–III**, several low electron density maxima were found which indicate the presence of additional disordered water molecules. These positions are not fully occupied and no reasonable structure model was found. Therefore, the data were corrected for disordered solvent using the SQUEEZE option in Platon.^19^ For compound **IV**, all of the water atoms are fully disordered and thus, these data were also corrected for disordered solvent using SQUEEZE. The crystal of **IV** was merohedrally twinned around a 2-fold axis and therefore, a twin refinement (twin matrix (01[combining macron]0) (1[combining macron]00) (001[combining macron])) was performed leading to a BASF parameter of 0.0297(9). One of the three independent Ni(en)_3_^2+^ counterions in **IV** exhibits slightly enlarged displacement parameters indicating some disorder. This complex is located on a special position, but if the refinement is performed in space groups of lower symmetry, the displacement parameters remain unchanged.

CCDC-1432847 (**I**), CCDC-; 1432848 (**II**), CCDC-; 1432849 (**III**), and CCDC-; 1432850 (**IV**) contain the supplementary crystallographic data for this paper.

### Magnetochemical characterisation

Magnetic data of **I–III** were recorded using a Quantum Design MPMS-5XL SQUID magnetometer. The polycrystalline samples were compacted and immobilised into cylindrical PTFE capsules. Data were acquired as a function of the field (0.1–5.0 T at 2 K) and temperature (2.0–290 K at 0.1 T). They were corrected for the diamagnetic contributions of the sample holder and the corresponding compound (**I–III**: *χ*_dia_ = –6.27 × 10^–4^ cm^3^ mol^–1^).

### Mass spectrometry

Electrospray ionisation quadrupole-time-of-flight high resolution mass spectrometric (ESI-Q-TOF-HRMS) experiments were performed with a Synapt G2-S HDMS (Waters Co., Milford, MA, USA) instrument. The flow rate was set to 10 μL min^–1^, the spray voltage to 1.6 kV, the sample cone voltage to 10 V, the source offset to 80 V, the nebuliser gas to 6 bar and the desolvation gas flow to 500 L h^–1^. Around these initial settings, the parameters were optimised for maximum abundance of the desired intact [M]^*n*–^ and [M·H_2_O]^*n*–^ cluster ions (*n* = 2, 3) and minimum abundance of fragments. For collision-induced dissociation (CID), N_2_ was used as the collision gas. Fragmentation experiments were conducted in the transfer cell of the Synapt G2-S HDMS instrument with collision energies of 15–25 V.

60 μM solutions from crystalline samples of **I** were prepared in H_2_O, D_2_O (Euriso-top, 99.90% D) and H_2_^18^O (Campro Scientific, 97% ^18^O), respectively. If not specified otherwise, the aqueous sample solutions were measured at this concentration after 30 min. Isotopic labelling experiments were accompanied by the corresponding control experiments in non-labelled H_2_O. Time-dependent measurements were conducted on samples kept at 4 °C from which aliquots were taken and directly subjected to the mass spectrometric experiments. All reaction times given therefore refer to the reaction at 4 °C.

## Results and discussion

### Syntheses

Compounds **I–III** crystallised from slurries of NH_4_VO_3_, Sb_2_O_3_, the corresponding transition metal chlorides and ethylenediamine at pH ≈ 14. According to the structural results, the V^V^ centres are reduced to V^IV^ which is a common observation, when such syntheses are performed in the presence of reducing amines. The formation of **I–III** is relatively insensitive against changes of the synthetic parameters. Compound **IV** could only be crystallised in the presence of 1-(2-aminoethyl)piperazine (aep). Originally, the synthesis with aep was performed to prepare antimonato POVs functionalised with an organic molecule as recently observed in the two compounds (C_6_H_17_N_3_)_2_[V_15_Sb_6_(C_6_H_15_N_3_)_2_O_42_(H_2_O)]·2.5H_2_O and [V_14_Sb_8_(C_6_H_15_N_3_)_4_O_42_(H_2_O)]·4H_2_O.^4^ The Ni source and en were added to enhance the structural diversity by an *in situ* formed complex. Surprisingly, the presence of aep in the reaction afforded crystallisation of **IV** as a (pseudo)polymorph of **I**.

### Characterisation and solubility studies

The IR spectra of **I–IV** (Fig. S1 and Table S1; ESI†) show the typical strong stretching vibration of the V^IV^=O group at around 960 cm^–1^. The DTA-TG curves of the samples are complex and exhibit no pronounced weight loss steps (Fig. S2, ESI†). For **I–III**, the total weight change observed up to *ca.* 250 °C corresponds to a loss of *ca.* 15 water molecules. Phase purity of the crystalline products was verified by X-ray powder diffraction (Fig. S3 and S4, ESI†). The crystal sizes and morphology were analyzed by SEM (Fig. S5, ESI†).

While solubility tests with less polar solvents such as alcohols, acetone, dichloromethane, chloroform and alkanes failed, compounds **I–IV** are surprisingly well soluble in distilled water. The maximum solubility in water has been determined for **I** to be 1.19 g L^–1^. The pH-value of a saturated solution increases from 6.5 (distilled water) to 8.2 (for details, see ESI†).

The product recovered after crystallisation from a saturated water solution was again characterised by X-ray powder diffraction, CHN analysis and IR spectroscopy. No significant changes were observed compared to the sample before dissolution. The starting material could thus be recovered unchanged, although with somewhat poorer crystallinity.

### Crystal structures

**Fig. 1:**
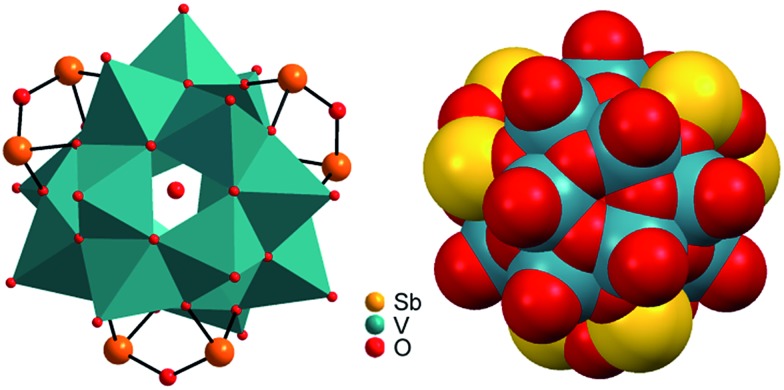
Left: Polyhedral representation of the [V_15_Sb_6_O_42_(H_2_O)_*x*_]^6–^ cluster anion. A part of the cluster anions contains encapsulated water molecules. Red: oxygen; orange: antimony. Right: Space-filling representation showing the cluster to be tightly closed so that the encapsulated water is trapped inside the cluster cavity.

**Fig. 2:**
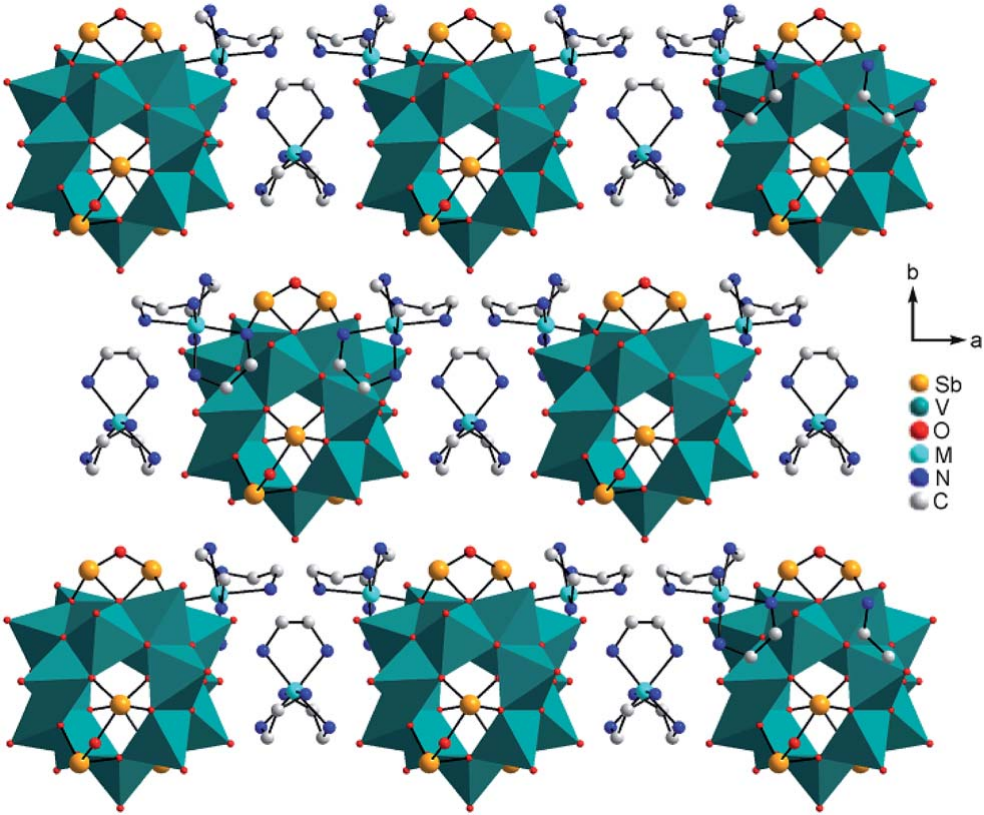
Arrangement of the cluster anions and cations in the structures of **I–III**. Hydrogen atoms and water molecules are not shown.

Compounds **I–III** crystallise in the chiral Sohncke space group *C*2 and **IV** in *P*321. Selected crystallographic data and refinement results are summarised in Table S2 (ESI†). In **I–III**, the three unique Sb atoms are on general positions, one of the eight crystallographically independent V atoms is located on a general position, and one of the three Ni centres is on a special position. In the structure of **IV**, the atoms V2, O1, O5, Ni1 and Ni3 are on special positions, whereas all other atoms are on general positions. All structures consist of isolated [V_15_Sb_6_O_42_(H_2_O)_*x*_]^6–^ (*x* = 0, 1) clusters with charge-compensating {M(en)_3_}^2+^ complexes. Residual electron density in the cluster cavities is consistent with single water molecules encapsulated in a fraction of the clusters. The cluster shell is constructed from 15 VO_5_ square pyramids sharing common edges and vertices and three Sb_2_O_5_ handles formed by corner-sharing of two SbO_3_ moieties (Fig. 1). The anion is structurally related to the {V_18_O_42_} archetype. Replacing three VO_5_ square pyramids by three Sb_2_O_5_ moieties yields the anions of the compounds under study here. The V–O bond lengths in the VO_5_ square pyramids are characterised by a short terminal V=O bond (*ca.* 1.6 Å) and four bonds to μ_3_-bridging O atoms (*ca.* 1.9–2.0 Å; Tables S3–S6, ESI†); as also observed in many POVs and chemically modified polyoxovanadates.^3^ The Sb–O bond lengths (1.9–2.0 Å) are typical for Sb^III^–O and match well those reported for other Sb-POVs.^5^,^20^ The cluster shells and {M(en)_3_}^2+^ counterions in **I–III** are arranged in a layer-like fashion with alternating anions and cations along all three axes (Fig. 2). The constituents in the structure of **IV** exhibit a similar arrangement (Fig. S7, ESI†). The oxidation states of Sb and V were determined as +III and +IV applying the bond valence sum method^21^ (BVS; see ESI† for details).

**Fig. 3:**
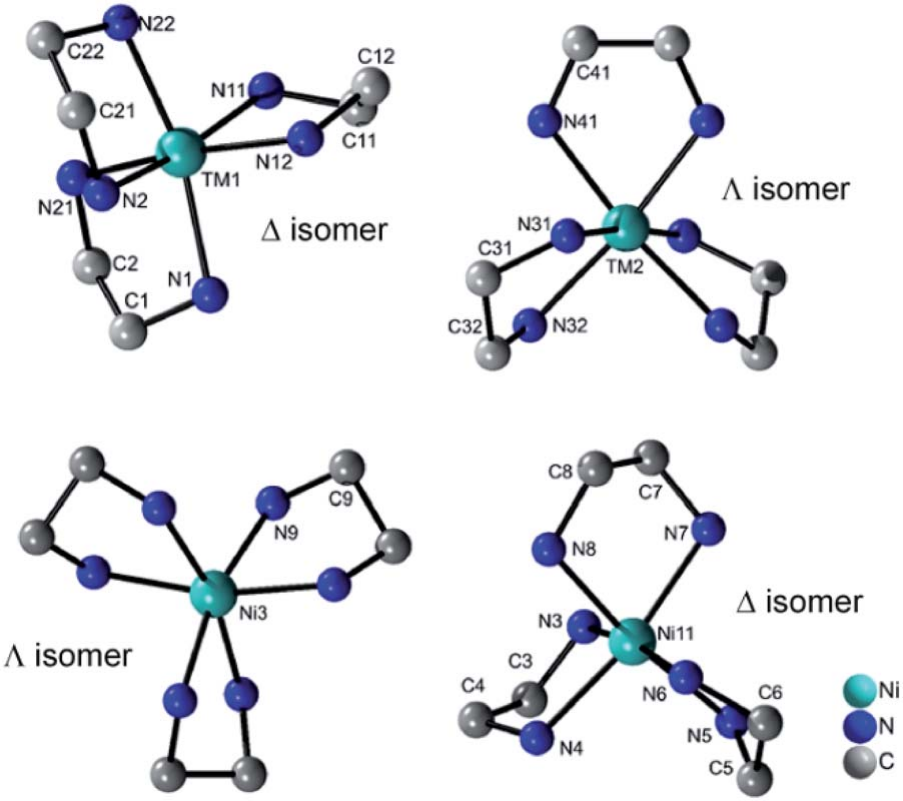
Molecular structures of the two different isomers of the {M(en)_3_}^2+^ cations in the structures of **I–III** (top) and of **IV** (bottom). H atoms are not displayed.

All four compounds contain three {M(en)_3_}^2+^ cations in distorted octahedral coordination geometries adopting the Δ and Λ isomer as configurations (Fig. 3). The M–N bond lengths (Tables S7–S10, ESI†) are in accordance with literature data.^22^ The distortion of the octahedra is evidenced by the N–M–N angles.

A dense hydrogen bonding network exists in the crystals between the NH hydrogen atoms of the ethylenediamine ligands and the oxygen atoms of the cluster anions (Tables S11–S14, ESI†). Not only terminal oxygen atoms are involved in H-bonding interactions, but also μ_3_-bridging O atoms. The ethylenediamine molecules also form hydrogen bonds to crystal water.

### Magnetic properties

**Fig. 4:**
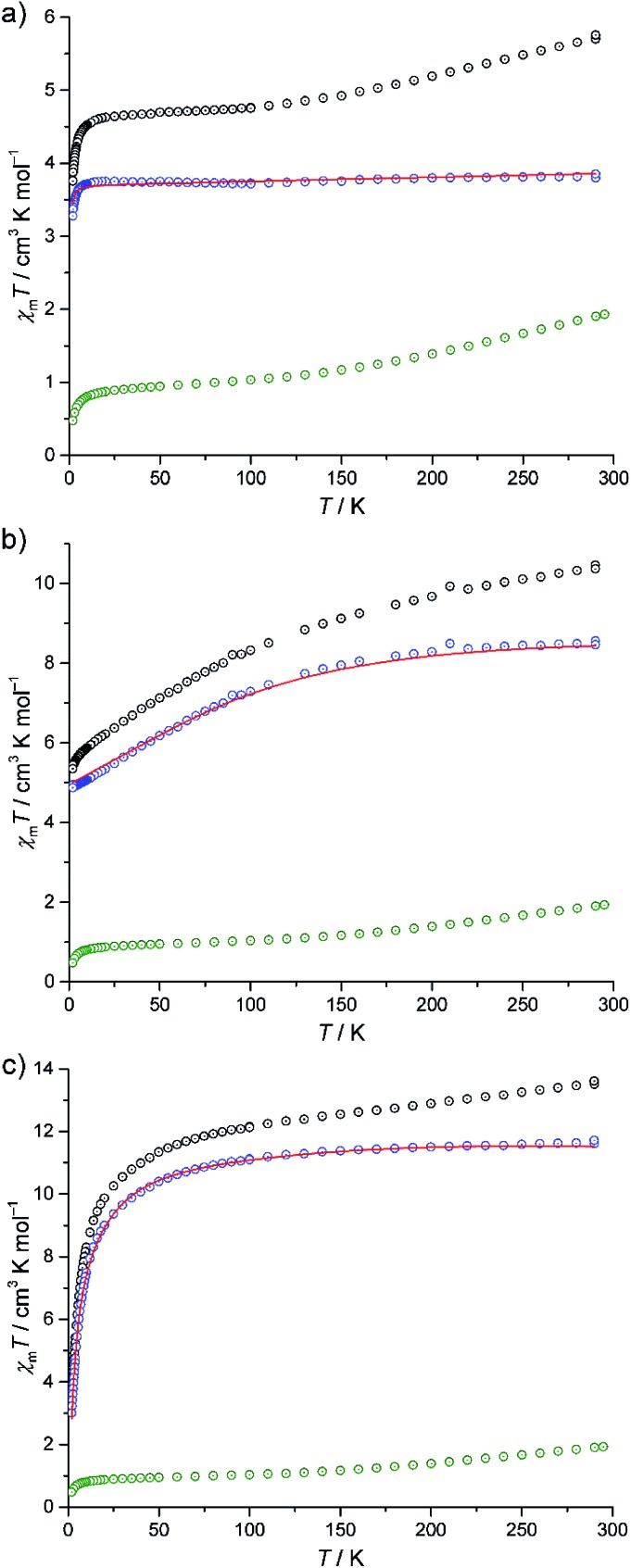
Temperature dependence of *χ*_m_*T* for (a) **I**, (b) **II** and (c) **III**. Black circles: experimental data; green circles: *χ*_m_*T*([V_15_Sb_6_O_42_(H_2_O)_*x*_]^6–^) (scaled); blue circles: difference of the experimental and [V_15_Sb_6_O_42_(H_2_O)_*x*_]^6–^ data. Red lines: least-squares fits.

The experimental magnetic susceptibility data for compounds **I–III** are shown in Fig. 4 as the temperature dependence of *χ*_m_*T* at 0.1 Tesla, and in Fig. S8 (ESI†) as molar magnetisation *M*_m_*vs.* magnetic field *B* at 2 K. The *χ*_m_*T vs. T* curves of all compounds are approximately linear from 290 K to 150 K. Aside from the differences in the absolute values of *χ*_m_*T*, the compounds show distinctly different *χ*_m_*T* evolution on further decrease in temperature, which primarily reflects the single-ion contributions of the different {M(en)_3_}^2+^ spin centres: for **I**, *χ*_m_*T* remains almost constant from 100 K to 20 K and subsequently decreases down to 3.76 cm^3^ K mol^–1^ at 2 K. For **II**, *χ*_m_*T* displays a steady decrease, reaching *χ*_m_*T* = 5.35 cm^3^ K mol^–1^ at 2 K. For **III**, the slope slightly decreases for 150 K ≥ *T* ≥ 100 K, and rapidly increases for lower temperatures, resulting in 3.48 cm^3^ K mol^–1^ at 2 K. At 290 K, the *χ*_m_*T* values of compounds **I–III** are above those expected for three non-interacting high-spin transition metal centres M (**I**: 5.72 cm^3^ K mol^–1^, expected:^23^ 2.94–4.60 cm^3^ K mol^–1^, **II**: 10.41 cm^3^ K mol^–1^, expected:^23^ 6.94–10.14 cm^3^ K mol^–1^, **III**: 13.58 cm^3^ K mol^–1^, expected:^23^ 9.76–12.19 cm^3^ K mol^–1^). On the other hand, these values are significantly below the expected values that are obtained by adding the contributions of 15 non-interacting V^4+^ centres, a consequence of the very strong antiferromagnetic coupling between the spin-1/2 vanadyl groups in the two outer V_6_ rings in {V_15_Sb_6_}.

Due to the rather large metal–metal distances and the absence of bridging ligands, the exchange interactions between the {M(en)_3_}^2+^ complexes and the POV cluster are expected to be negligible. Since the susceptibility data for the isolated {V_15_Sb_6_} cluster are known,5*a* a first estimation of the magnetic properties of the {M(en)_3_}^2+^ complexes within the compounds can be obtained by the following subtraction method. We use **I** as the reference system, since an octahedrally coordinated Ni^2+^ (d^8^) centre can be treated as a spin-only system due to the orbital singlet ground term ^3^*A*_2_, resulting in nearly temperature-independent *χ*_m_*T* values. *χ*_m_*T* data of **I** are subtracted by scaled *χ*_m_*T* data of [V_15_Sb_6_O_42_(H_2_O)_*x*_]^6–^, yielding a curve (Fig. 4a, blue circles) corresponding to the expected single-ion contributions of three spin-1 Ni^2+^ centres. The thus-determined scaling factor of *ca.* 0.9 reflects differences in the amount of crystal solvents and the cationic lattice. The corresponding scaled contribution for the individual {V_15_Sb_6_} polyoxoanion is shown in Fig. 4a–c as green circles for reference. The same subtractive method, employing the same scaling factor, is then applied to compounds **II** (Co^2+^, d^7^) and **III** (Fe^2+^, d^6^) in which ligand field effects dominate the lower temperature behaviour of *χ*_m_*T*.

Our computational framework CONDON 2.0,^24^ employing a “full model” Hamiltonian has been used to model the post-subtraction susceptibility data of **I–III** (Fig. 4 and S8,† open blue circles). Since the octahedral site symmetry is slightly distorted, a single additional ligand field parameter *B*_0_^2^ (with respect to perfect octahedral symmetry *O*_h_) is introduced which reflects *C*_4v_ site symmetry. The mean field parameter *zJ*′ representing potential exchange interactions is allowed to vary to test the hypothesis of negligible exchange interactions. The least-squares fits (of moderate goodness-of-fit, SQ ≈ 2% for **I–III**) are shown as red lines in Fig. 4 and S8 (ESI†), and the corresponding model parameters are given in Table S15 (ESI†). We emphasise that the subtraction method employed here can only be understood as a first approximation and the fit parameters should be interpreted accordingly. The ligand field parameters represent a ligand field of distorted octahedral symmetry, and a ligand field splitting of 10 Dq approximately 10 000 cm^–1^ (**I**), 16 000 cm^–1^ (**II**), and 7000 cm^–1^ (**III**). Within the limits of method, the small mean field parameters *zJ*′ (**I**: –0.01 cm^–1^, **II**: +0.01 cm^–1^, **III**: –0.53 cm^–1^) are in agreement with virtual absence of exchange interactions between the transition metals of the {M(en)_3_}^2+^ complexes and neighbouring POV groups in the solid state. The different signs should also be understood as remnants of the subtraction method, as the absolute *zJ*′ values are very small. Therefore, the apparent temperature dependences of *χ*_m_*T* of **II** and **III** are not a consequence of potential exchange but due to ligand field effects within the {M(en)_3_}^2+^ complexes.

### Electrospray ionisation mass spectrometry

**Fig. 5:**
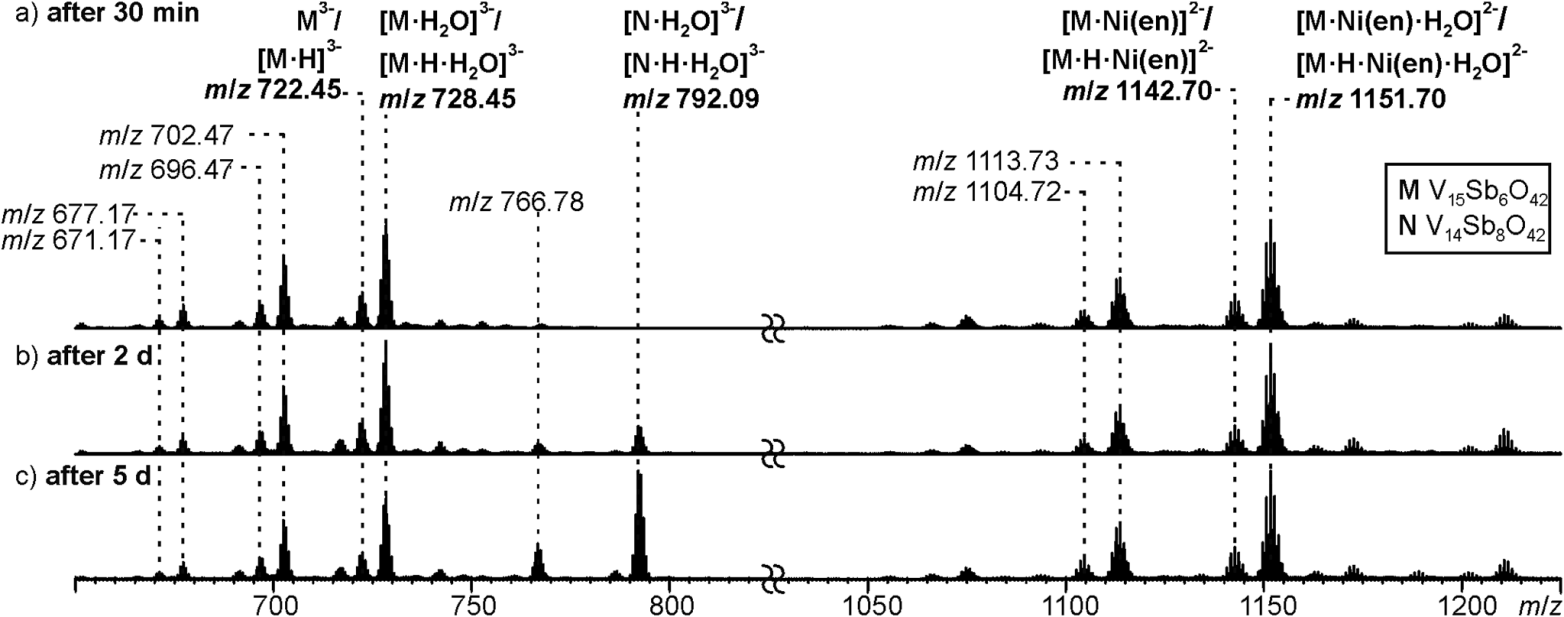
ESI-Q-TOF-HRMS spectra of compound **I** (60 μM in H_2_O) recorded from the same sample after (a) 30 minutes, (b) 2 days, (c) 5 days. For a more detailed discussion of the non-labeled signals, see ESI.†

**Fig. 6:**
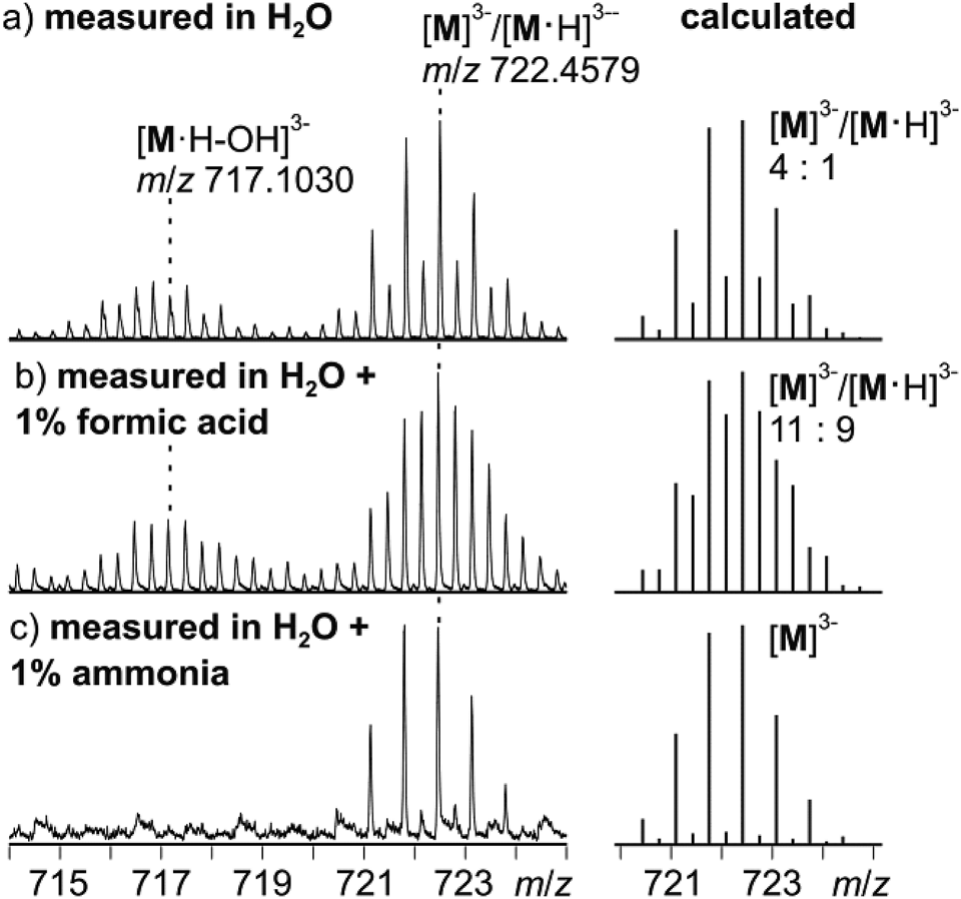
Left: Experimental isotopic patterns of the trianion at *m*/*z* 722 as obtained from 60 μM solutions of **I** in (a) H_2_O, (b) H_2_O + 1% formic acid and (c) H_2_O + 1% ammonia. Right: Calculated isotopic patterns of **M**^3–^/[**M**·H]^3–^ mixtures with compositions fitted to approximate the experimental isotopic patterns.

Negative-mode electrospray ionisation of a 60 μM water solution of compound **I** 30 min after dissolving the crystalline sample results in the ESI mass spectrum shown in Fig. 5a. Two very similar series of signals are observed, one with doubly (*m*/*z* 1050–1300) and one with triply charged anions (*m*/*z* 650–800). Signals of the intact cluster appear as the triply charged [V_15_Sb_6_O_42_]^3–^ (**M**^3–^) ion at *m*/*z* 722 and its complex [**M**·Ni(en)]^2–^ at *m*/*z* 1141. Overall, the cluster core in the crystalline sample is a hexaanion. As it appears in the mass spectrum at *m*/*z* 722 as a triply charged ion, three one-electron oxidation steps of V^IV^ to V^V^ have taken place, likely induced by the high voltage of the electrospray needle and supported by significant charge repulsion among the six charges in the absence of stabilizing counterions and solvent molecules after ionisation. As the Ni(en)^2+^ fragment is doubly charged, the cluster core must be tetraanionic in the ion at *m*/*z* 1141 (**M**^4–^). This indicates that higher charge states can form even in the gas phase, when stabilizing counterions are present. A detailed look at the isotopic pattern of the *m*/*z* 722 trianion (Fig. 6a) reveals that actually two patterns overlap that are shifted against each other by Δ*m*/*z* = 0.33. They can be assigned to [V_15_Sb_6_O_42_]^3–^ (**M**^3–^) and [HV_15_Sb_6_O_42_]^3–^ ([**M**·H]^3–^), the latter of which also contains a quadruply charged cluster core with one charge compensated by a proton. From the calculated isotope patterns of these two ions, the experimental one can be simulated and one obtains a 4 : 1 ratio of the two ions when sprayed from pure water solution. As controls, the same experiments were repeated with 1% of formic acid (Fig. 6b) and 1% of ammonia (Fig. 6c) added to the sample solution. Clearly, the ratio of the two ions is shifted towards protonated cluster under acidic and to the non-protonated one under basic conditions. This confirms the peak assignment. The signal at *m*/*z* 722 is accompanied by a somewhat smaller signal at *m*/*z* 717, which can be assigned to [**M**·H–OH]^3–^. As this signal completely vanishes together with the protonated trianion cluster under basic conditions (Fig. 6c), it is very likely due to a fragmentation of [**M**·H]^3–^ during ionisation. The other signal at *m*/*z* 728 (Fig. 5a) corresponds to a superposition of the two water adducts [**M**·H_2_O]^3–^ and [**M**·H·H_2_O]^3–^. Similar considerations also apply to the doubly charged ions.

### Cluster reactivity with water

In order to investigate the stability of **I** in water, the mass spectrometric experiments were repeated after longer reaction intervals up to five days (Fig. 5b and c). Over time, a new signal appears at *m*/*z* 792 which can be assigned to [V_14_Sb_8_O_42_·H_2_O]^3–^ ([**N**·H_2_O]^3–^) and [**N**·H·H_2_O]^3–^, an Sb-richer cluster formed by a net exchange of a V=O against an Sb–O–Sb unit. This rearrangement reveals an astonishing reactivity; especially when taking into account that all {V_14_Sb_8_O_42_} clusters known so far were prepared under solvothermal conditions. From these findings, we conclude that the comparably high solubility of compound **I** enables its use for post-functionalisation into other cluster compounds. At longer reaction times, also a visible precipitate forms, which we attribute to the corresponding [V_16_Sb_4_O_42_(H_2_O)_*x*_] product cluster that is expected to be cogenerated in a V=O against Sb–O–Sb exchange reaction.

### H/D-exchange experiments

**Fig. 7:**
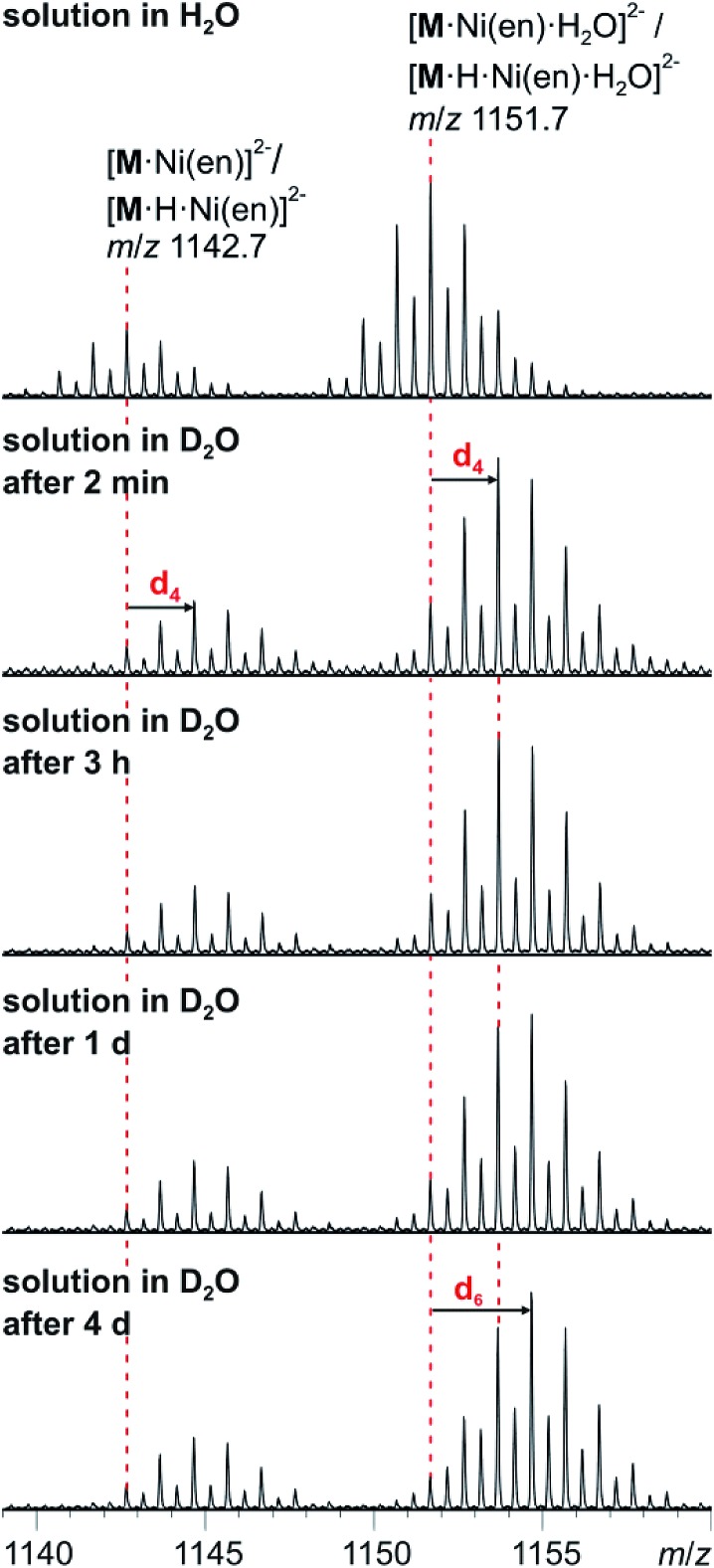
H/D-exchange experiment. ESI-MS measurements with compound **I** (60 μM solution in D_2_O) after different reaction intervals.

The solution-phase exchange of labile hydrogen atoms against deuterium can be followed by ESI mass spectrometry, when cluster **I** is dissolved in D_2_O and then sprayed after different reaction times. The exchange of the ethylenediamine NH atoms is expected to be fast and should be easily monitored for the doubly charged ions [**M**·Ni(en)]^2–^ and [**M**·Ni(en)·H_2_O]^2–^as they still contain one ethylenediamine ligand with four N-centred hydrogen atoms. Indeed, a complete exchange of these four hydrogen atoms occurs instantly for all en-containing ions (Fig. 7). Already after two minutes, a shift of the isotope patterns of [**M**·Ni(en)]^2–^ and [**M**·Ni(en)·H_2_O]^2–^ by Δ*m*/*z* = 2 is observed indicating that all four NH hydrogen atoms have been fully exchanged. Remarkably, the exchange of the two water hydrogen atoms in the water adduct [**M**·Ni(en)·H_2_O]^2–^ is very slow and proceeds only over days (Fig. 7). This result is not in agreement with an intact cluster structure that is incompletely desolvated during ionisation. Any weakly bound solvent water molecule in the cluster periphery should be replaced by a D_2_O immediately, when the cluster is sprayed from deuterated water. Therefore, the H_2_O molecule must be an integral part of the cluster structure in solution. Two possibilities exist: either the water molecule has added to and opened one of the oxo-bridges resulting in a structure bearing two OH groups in the cluster shell or one water molecule is encapsulated inside the cavity of the closed cluster. As one would certainly expect, the metal ion-centred OH groups to be acidified by the metal ion, the dihydroxy structure is also expected to undergo a fast H/D-exchange reaction. Consequently, the only structure of the water adduct, which is in agreement with the slow H/D-exchange, is a closed capsule with a single water ion inside. This structure is not only in agreement with the fact that a potential adduct containing two water molecules has never been observed, but is also in agreement with the residual electron density observed in the crystal structure (see above). Given the tightly closed cluster shell (Fig. 1, right), a slow exchange of the intact encapsulated water molecule through portals in the cluster walls can be ruled out. We thus rationalise the finding of the slow H/D-exchange as follows: the inner-phase water attacks one of the vanadium ions as a weak nucleophile. This step is followed by opening one of the oxo-bridges so that two OH groups exist, which can undergo the H/D-exchange reaction. One of the initial steps (attack of the vanadium ion or oxo-bridge opening) is rate-determining so that the exchange is slow, even when the exchange of the hydrogen atoms in the open, dihydroxy intermediate is fast. Back reaction after the exchange leads back to the closed capsule with a water molecule residing in the cavity. These experiments thus demonstrate that the water-filled cluster under study exhibits quite remarkable inner-phase reactivity that can be monitored by ESI-MS.

### Tandem MS experiments

MS/MS experiments were performed by first mass-selecting either one of the complexes **M**^3–^/[**M**·H]^3–^, [**M**·H_2_O]^3–^/[**M**·H·H_2_O]^3–^, **N**^3–^/[**N**·H]^3–^ and [**N**·H_2_O]^3–^/[**N**·H·H_2_O]^3–^ (*m*/*z* 722, 728, 786, 792) and subsequently subjecting them to collision-induced dissociation (CID). Note that the intensity of the pure **M**^3–^ trianion obtained, when ammonia is added to the sample solution, is too low for this experiment. Therefore, the experiments have been conducted with the overlapping clusters.

**Fig. 8:**
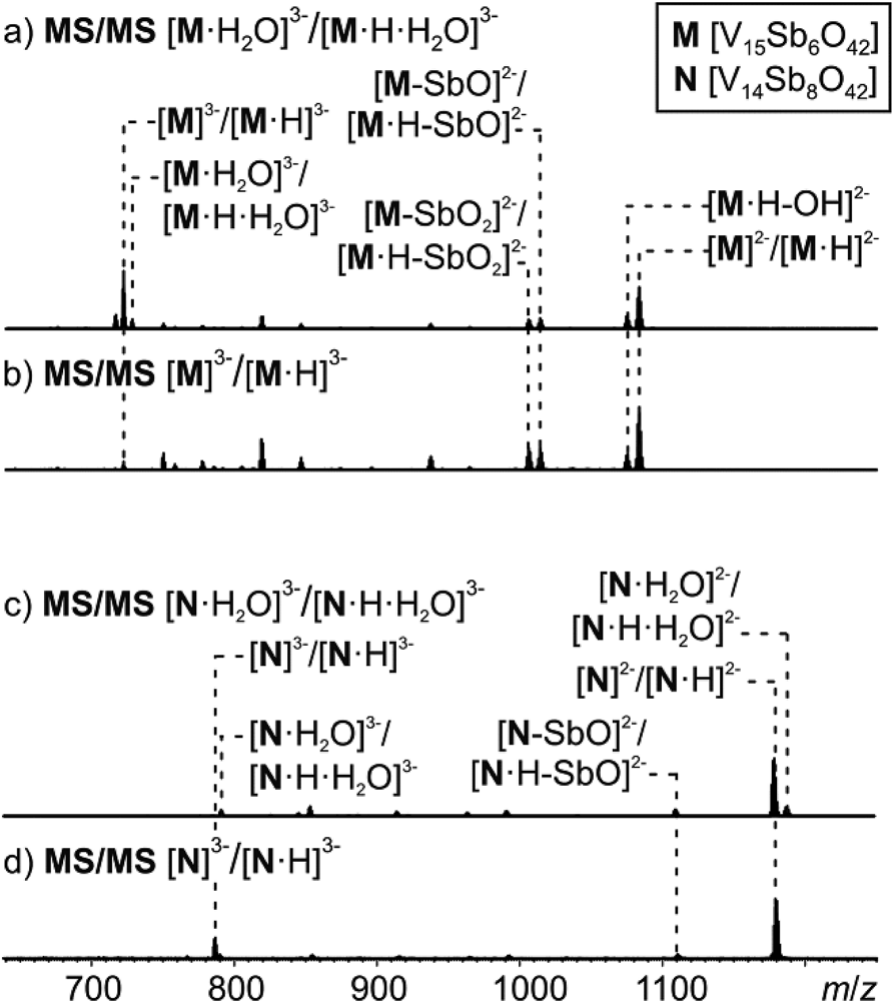
Tandem MS experiment of (a) complex [**M**·H_2_O]^3–^, (b) complex **M**^3–^, (c) complex [**N**·H_2_O]^3–^, (d) complex **N**^3–^.

The fragmentation of the clusters encapsulating water begins with a loss of a water molecule yielding **M**^3–^/[**M**·H]^3–^ and **N**^3–^/[**N**·H]^3–^, respectively (Fig. 8a and c). All subsequent fragmentation reactions are qualitatively the same as those observed for mass-selected **M**^3–^/[**M**·H]^3–^ and **N**^3–^/[**N**·H]^3–^ generated in the ion source (Fig. 8b and d): loss of a hydroxyl radical from the protonated clusters and electron losses to yield the corresponding doubly charged clusters **M**^2–^/[**M**·H]^2–^ and **N**^2–^/[**N**·H]^2–^. Subsequent losses of SbO units and further fragmentation of the cluster core produce the other fragments observed.

Most interestingly, the water loss from [**M**·H_2_O]^3–^/[**M**·H·H_2_O]^3–^ is clearly less energy demanding than the electron loss as no [**M**·H_2_O]^2–^/[**M**·H·H_2_O]^2–^ ions are visible in the spectrum in Fig. 8a. Its activation barrier must consequently be smaller than the (unfortunately unknown) electron affinity of the cluster dianion in the gas phase. However, the ions [**M**·H–OH]^3–^ (very small signal) and [**M**·H]^2–^ appear simultaneously as fragments from [**M**·H]^3–^. This implies that the electron and OH losses compete and thus are similar in energy. Consequently, the water loss from [**M**·H·H_2_O]^3–^ has a lower barrier than the OH loss from [**M**·H]^3–^.

**Scheme 1:**
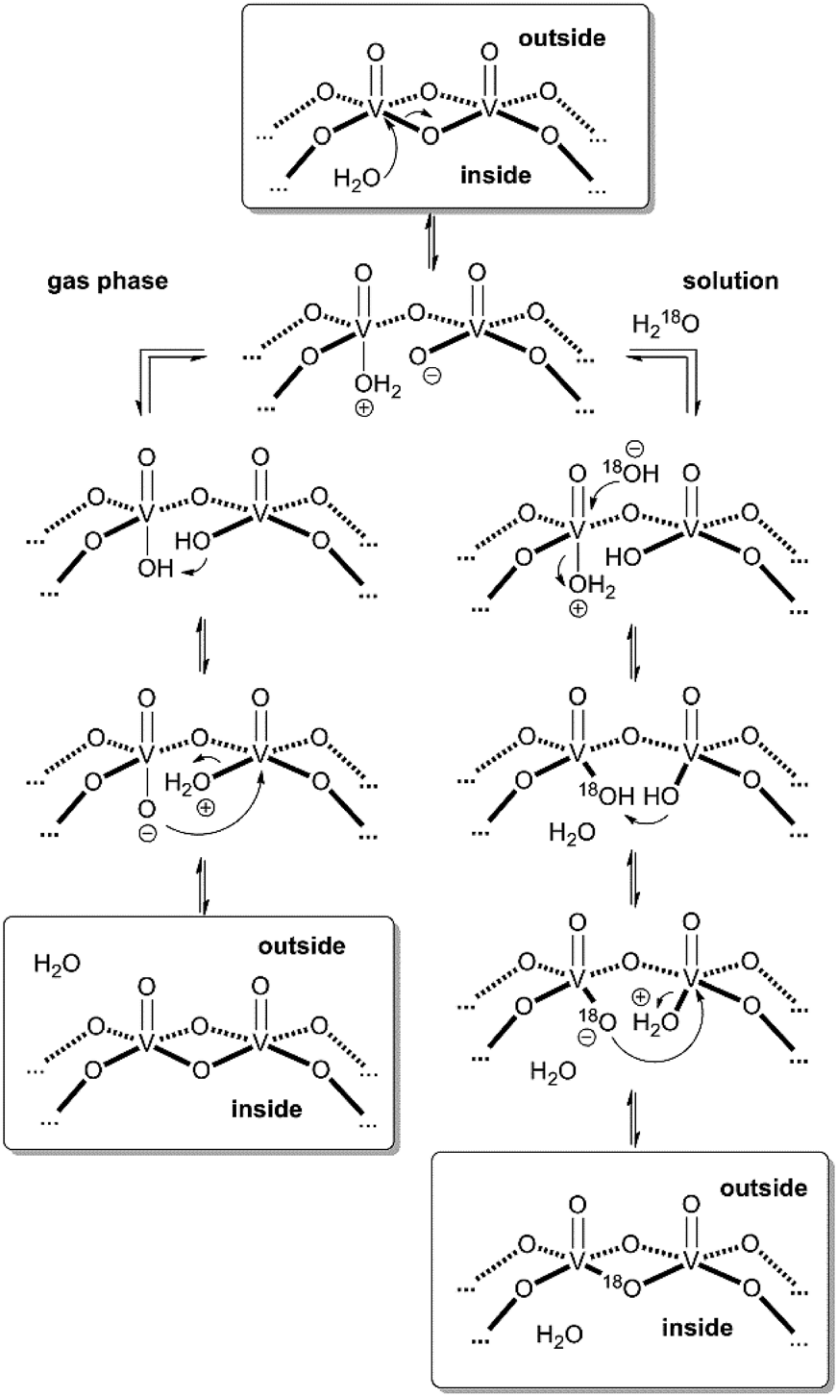
“Swinging door” mechanism for the water loss from [**M**·H_2_O]^3–^ in the gas phase (left) and mechanism for the solution-phase ^16^O/^18^O exchange in cluster **I** (right).

These considerations render mechanisms for the water loss from [**M**·H·H_2_O]^3–^ unlikely which involve the simultaneous cleavage of more than one metal–oxygen bond. Therefore, we suggest the “swinging-door” mechanism shown in Scheme 1 (left) for the H_2_O loss. The encapsulated water molecule initially attacks one of the vanadium ions and – as postulated above to rationalise the H/D-exchange – opens one of the oxo-bridges. After two proton transfer steps, the bridge closes again, this time however with a water molecule as the leaving group that is lost on the outside of the cluster. The water loss in the gas phase is entropically favourable due to particle number increase. Both the H/D-exchange reaction in water solution and the water loss mechanism in the gas phase thus share common elementary steps.

### ^16^O/^18^O exchange experiments

**Fig. 9:**
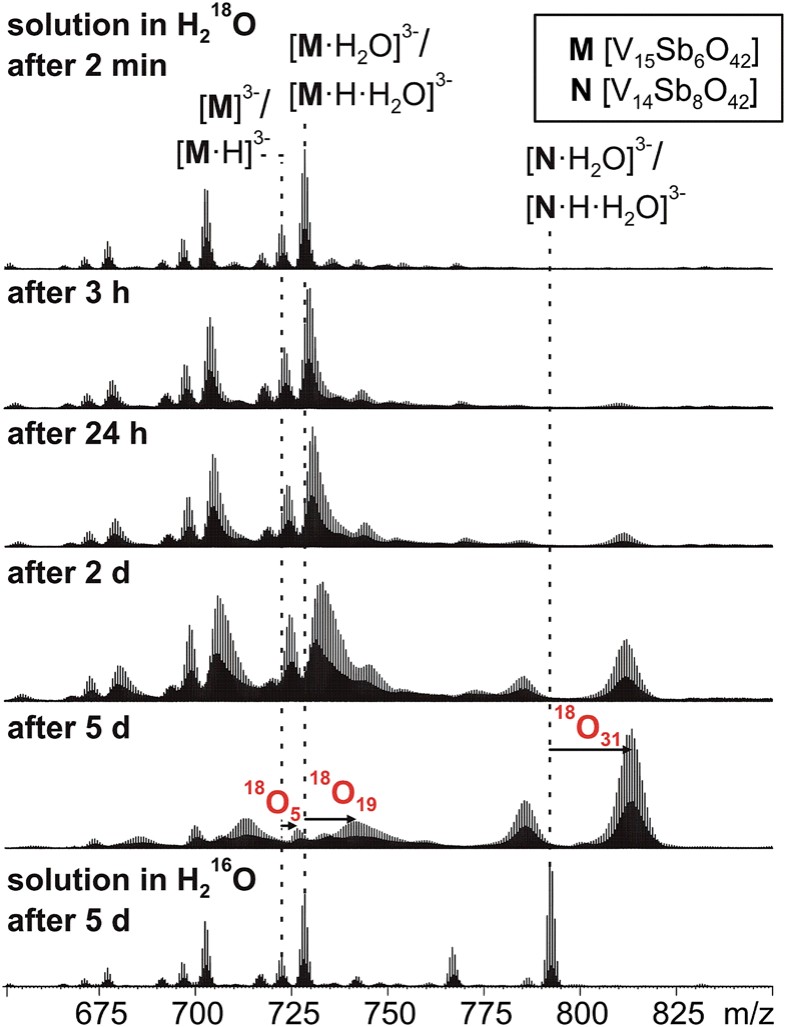
^16^O/^18^O exchange experiments performed with a 60 μM solution of **I** in H_2_^18^O after different reaction intervals.

When a sample of cluster **I** was prepared in H_2_^18^O, an exchange of ^16^O against the water ^18^O atoms is observed (Fig. 9). Again, the exchange is slow and proceeds over days being in agreement with further investigations of Murman *et al.*^25^ However, most strikingly, all cluster ions with encapsulated water undergo a much faster ^16^O/^18^O exchange reaction than the water-free cluster ions. Even though a detailed kinetic fitting of the data is not straightforward because of overlapping isotope patterns and the need to apply different rate constants for different types of oxygen atoms in the cluster structure, the acceleration is easily seen qualitatively in the spectra after a reaction time of five days: the shift of the maximum of the isotopic patterns of water-free **M**^3–^/[**M**·H]^3–^ corresponds to the exchange of only five oxygen atoms, while the isotopic patterns of the corresponding water-containing cluster ions [**M**·H_2_O]^3–^ and [**M**·H·H_2_O]^3–^ have shifted by the equivalent of 19 oxygen atom exchanges.

This finding leads to several conclusions: (i) the interpretation of the H/D-exchange experiments is confirmed in that two distinctly different structures exist in solution – the cluster with and that without encapsulated water. The water molecule is thus not a solvate water. (ii) The two structures with and without encapsulated water do not interconvert quickly on the time scale of the ^16^O/^18^O exchange experiment. Otherwise, the remarkable rate differences between the two cluster ions would not be observed. (iii) As the only difference between the two ions is the absence/presence of encapsulated water, the inner-phase water molecule clearly has a significant effect on the outer-phase reactivity. The information that the water molecule is present inside is thus transduced through the cluster shell and influences the cluster's reactions with the surrounding environment.

The post-functionalised V=O-to-Sb_2_O exchange product appears as the [**N**·H_2_O]^3–^/[**N**·H·H_2_O]^3–^ trianion pair and has undergone even more ^16^O/^18^O exchange steps (31 after 5 days). Very likely, some intermediates encompassed during the metal ion exchange reactions are not fully saturated and thus can exchange ^16^O against ^18^O even faster.

Based on the mechanistic considerations above, we suggest the ^16^O/^18^O exchange to proceed through similar initial steps. The acceleration of this reaction can easily be rationalised by invoking again an attack of the encapsulated water at one of the vanadium ions followed by oxo-bridge cleavage, the exchange reaction and reformation of the cluster which then incorporates an ^18^O atom (Scheme 1, right). In contrast to the gas phase, the cluster is now surrounded by water so that an escape of the encapsulated water from the cavity is neither favoured by entropy (exclusion volume inside the cavity) nor enthalpy (non-solvated inner surface of the cluster). As the inner-phase water molecule is reformed after the ^16^O/^18^O exchange reaction and thus able to accelerate many exchange reactions, one can consider it a catalyst.

## Conclusions

Four new heteroatom-modified polyoxovanadate compounds of the general composition{M(en)_3_}_3_[V_15_Sb_6_O_42_(H_2_O)_*x*_]·*n*H_2_O (M = Fe^II^, Co^II^, Ni^II^) were synthesised under solvothermal conditions and characterised by a combination of complementary methods including crystallography and magnetic property measurements. The Ni compound crystallises in two pseudopolymorphs depending on the reaction conditions. Its unexpectedly high solubility in water makes it a perfect candidate for post-functionalisation studies which provide access to new polyoxovanadate clusters. While this strategy is well known for polyoxomolybdates and polyoxotungstates, the often limited solubility of larger polyoxovanadates so far hampered such an approach. The magnetic properties of the compounds can be rationalised by a qualitative model of additive contributions by strongly antiferromagnetically coupled {V_15_Sb_6_} cluster units and the three high-spin {M(en)_3_}^2+^ complexes, with virtually no exchange coupling between those groups. In line with crystallography, the electrospray ionisation mass spectrometric experiments reveal that a large fraction of the clusters contains encapsulated water which is protected in solution against a fast H/D-exchange by the cluster shell. This inner-phase water molecule participates in the cluster's reactivity as it can accelerate oxo-bridge opening reactions by attacking a vanadium ion from the inside of the cluster cavity. Consequently, the water molecule inside the cavity displays inner-phase reactivity. Most fascinatingly, its presence also catalyses ^16^O/^18^O exchange reactions between the cluster shell and the surrounding water. Thus, the inner-phase reactivity of the encapsulated water has a significant effect on the outer-phase reactivity of the cluster as well. A transduction of the information that a water molecule is present inside thus affects the reactivity of the cluster periphery.

## Supplementary Material

Supplementary information

Crystal structure data
