# Supplementary material for: Catalysis of “outer-phase” oxygen atom exchange reactions by encapsulated “inner-phase” water in {V15Sb6}-type polyoxovanadates
Source: Chem Sci. 2016 Jan 8;7(4):2684–94. doi: 10.1039/c5sc04571a (PMC5477048; doi:10.1039/c5sc04571a)
Supplement: Supplementary file 1 [file SC-007-C5SC04571A-s001.pdf]

Electronic Supplementary Material (ESI) for Chemical Science.
This journal is © The Royal Society of Chemistry 2016

Electronic Supplementary Information

Catalysis of "Outer-Phase" Oxygen Atom Exchange Reactions by Encapsulated "Inner-Phase" Water in $\{V_{15}Sb_6\}$-type Polyoxovanadates

Michael Wendt[a,*], UlrikeWarzok[b,*], Christian Näther[a], Jan van Leusen[c], Paul Kögerler[c], Christoph A. Schalley[c,#], Wolfgang Bensch[a,#]

[a] *Institut für Anorganische Chemie, Christian-Albrechts-Universität zu Kiel, Max-Eyth-Str. 2, 24118 Kiel, Germany*

[b] *Institut für Chemie und Biochemie der Freien Universität, Takustr. 3, 14195 Berlin, Germany*

[c] *Institut für Anorganische Chemie, RWTH Aachen, Landoltweg 1, 52074 Aachen, Germany*

Contents

Spectroscopic Characterization

Crystallographic Data

Magnetic Properties

Electrospray Mass Spectrometry

Figure S1 IR spectra of compounds **I** – **III** S2

Table S1 Assignment of the IR peaks of compounds **I** – **III** S2

Figure S2 TG curve of **I** S3

Figures S3 + S4 Powder Diffraction Patterns of **I** – **III** and **IV** S4-5

Figure S5 SEM pictures of all new compounds **I** – **IV** S6

Figure S6 UV/Vis Absorption spectra for Solubility Studies S7

Table S2 – S6 Crystallographic Data S8-11

Figure S7 Arrangement of the cluster anions and transition metal complex cations of **IV** S12

Table S7 – S10 Bond length and angles of the transition metal amine comples in **I** – **IV** S12-S17

Table S11 – S14 Hydrogen interactions in **I** – **IV** S18-S19

Figure S8 Molar magnetization M_m as a function of the applied field B S20

Table S15 Parameters of the "full model" simulations S21

Electrospray Mass Spectrometry S22

References S23

1. IR Spectra

In Figure S1, the IR spectra of compounds **I** – **III** are displayed. As compound **IV** is a (pseudo)polymorph of **I**, both have equal IR spectra. All bands were assigned to the organic molecules or to the cluster vibrations. The values and assignments are listed in Table S1.

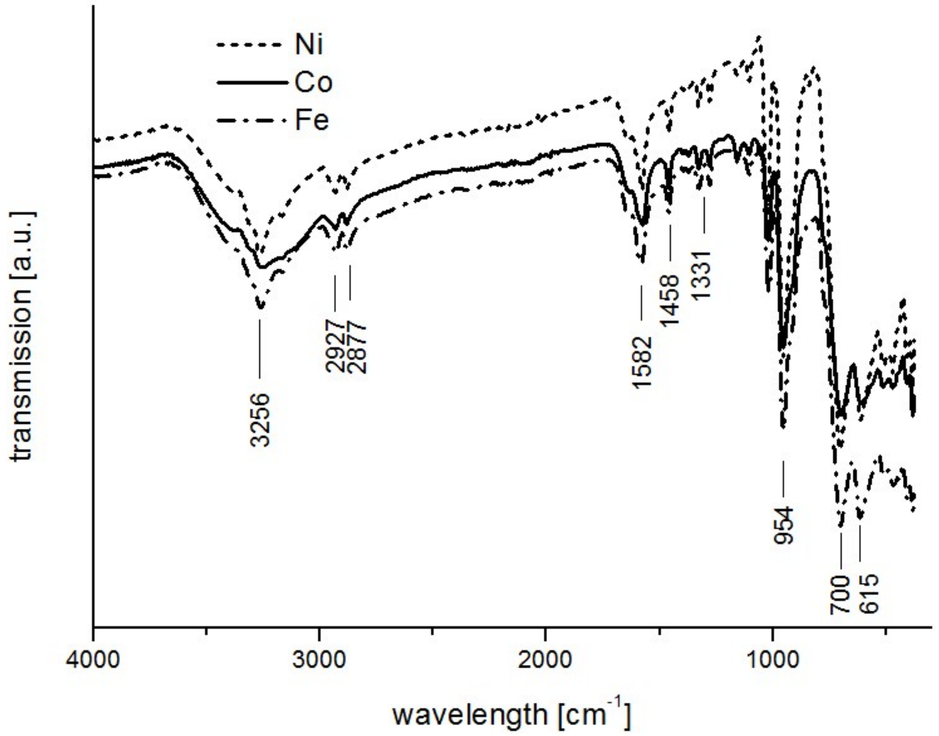

Figure S1. IR spectra of compounds **I** – **III**.

Table S1 Assignment of the IR peaks of compounds **I** – **III**.

	**wavenumber [cm^{-1}]**		**assignment**
Compound **I**	Compound **II**	Compound **III**	
3256	3257	3265	crystal water, NH_2-stretch
2927	2933	2934	CH_2 stretch
2877	2877	2884	
1582	1575	1575	NH_2 deformation
1458	1459	1460	CH_2 deformation
1331	1331	1331	OH deformation
954	953	954	V^{IV}=O stretch
700	696	701	M-O-M stretch
615	611	613	M-O-M stretch

2. Thermogravimetric Analysis

Figure S2 shows a DTA-TG curve of compound **I** as a representative example. For all compounds, first a weight loss due to water emission is observed being in accordance with the presence of ca. 15 water molecules in compounds **I** – **III**. The different not well resolved mass steps cannot be assigned to individual decomposition reactions. For compound **IV**, the first mass loss was ≈ 13 % and corresponds to the emission of ca. 28 water molecules.

Previous DTA-TG experiments on Sb-POVs[1] displayed analogous behavior resulting in no distinct steps for the decomposition and the weight loss above 700 °C is assigned to the sublimation of antimony, which is in agreement with our measurements.

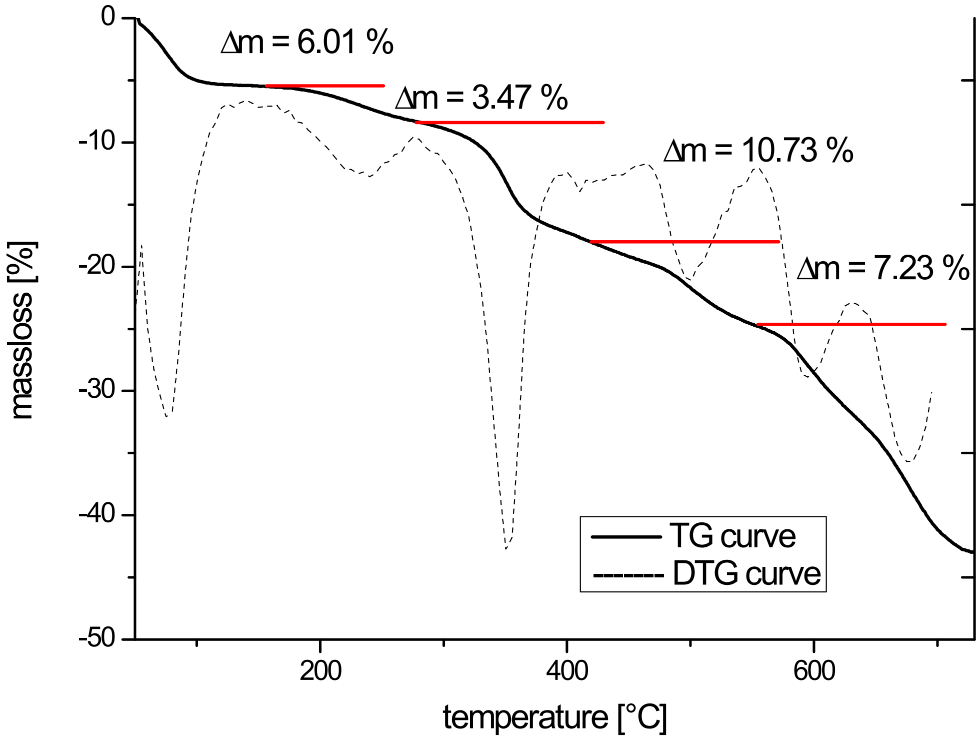

Figure S2. TG-curve (black) and DTG-curve (dotted) of compound **I** ($\{Ni(en)_3\}_3 [V_{15}Sb_6O_{42}(H_2O)]\bullet xH_2O$ (heating rate: 1 K min^{-1}).

3. Powder Diffraction Patterns

Figures S3 and S4 indicate phase purity of all four compounds **I** – **IV** as evidenced by a comparison of experimental powder patterns with calculated patterns using single crystal data. Both reflection intensities and 2θ angles are in excellent agreement with the calculated diffractions.

a)

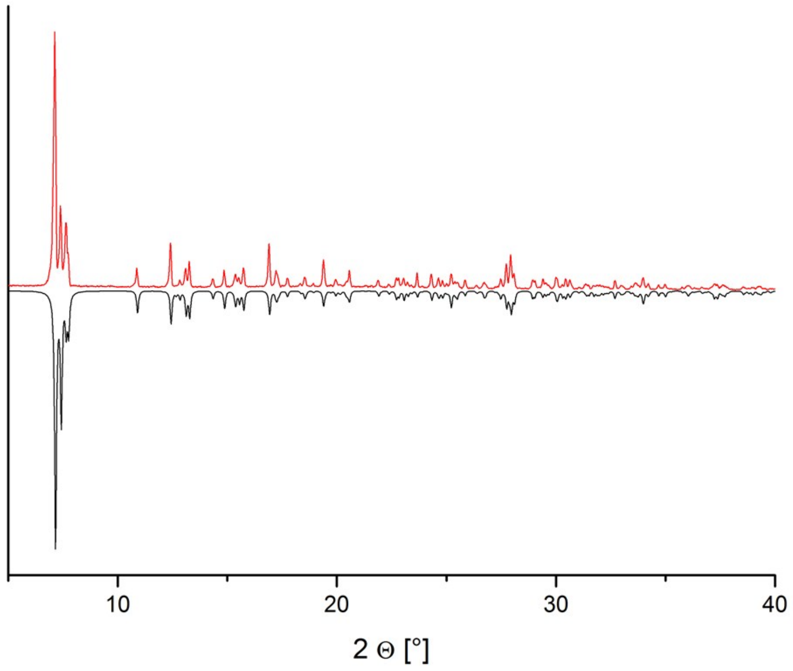

b)

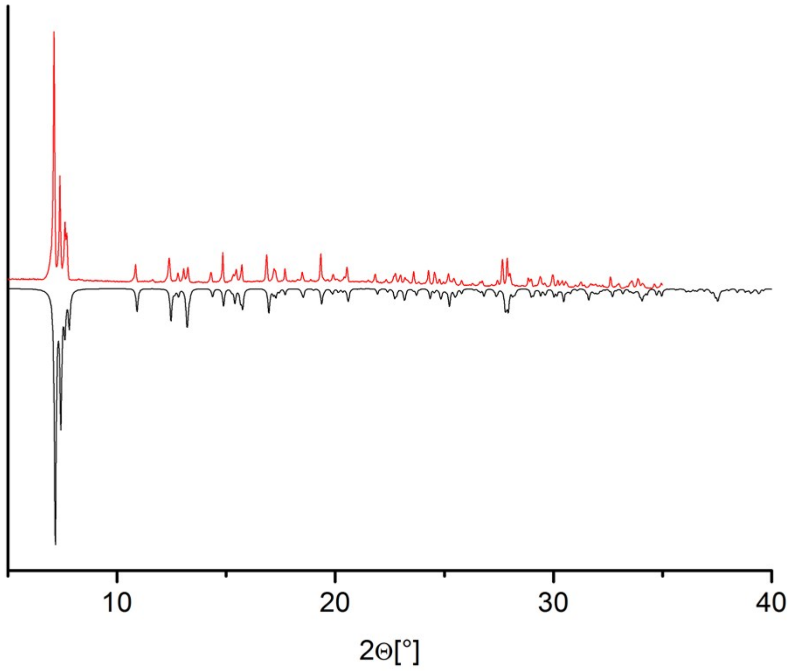

c)

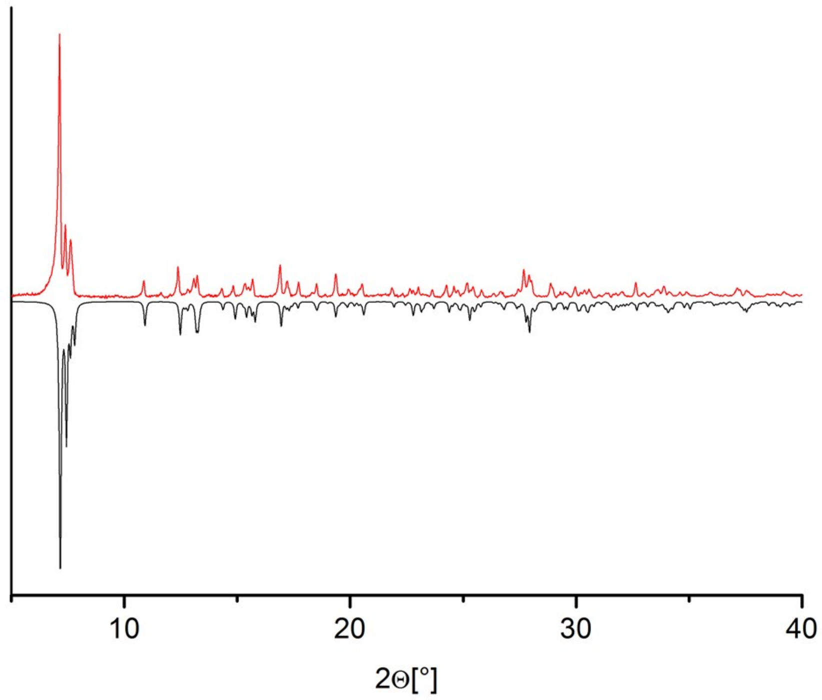

Figure S3. Measured (red) and calculated (black) powder diffraction patterns of compound **I**.

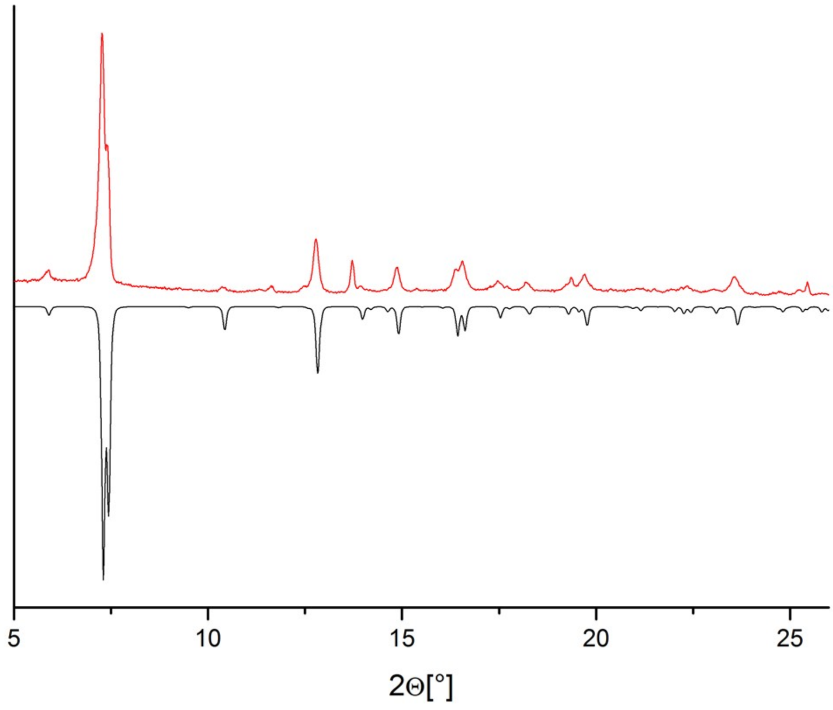

Figure S4. Measured (black) and calculated (red) powder diffraction patterns of compound **IV**.

4. Crystal Morphology

Figure S5 shows the morphology of the crystals of compounds **I** - **IV**. The crystal sizes could be determined to be around 1200 x 200 µm for **I**-**III** and 1000 x 125 µm for **IV**.

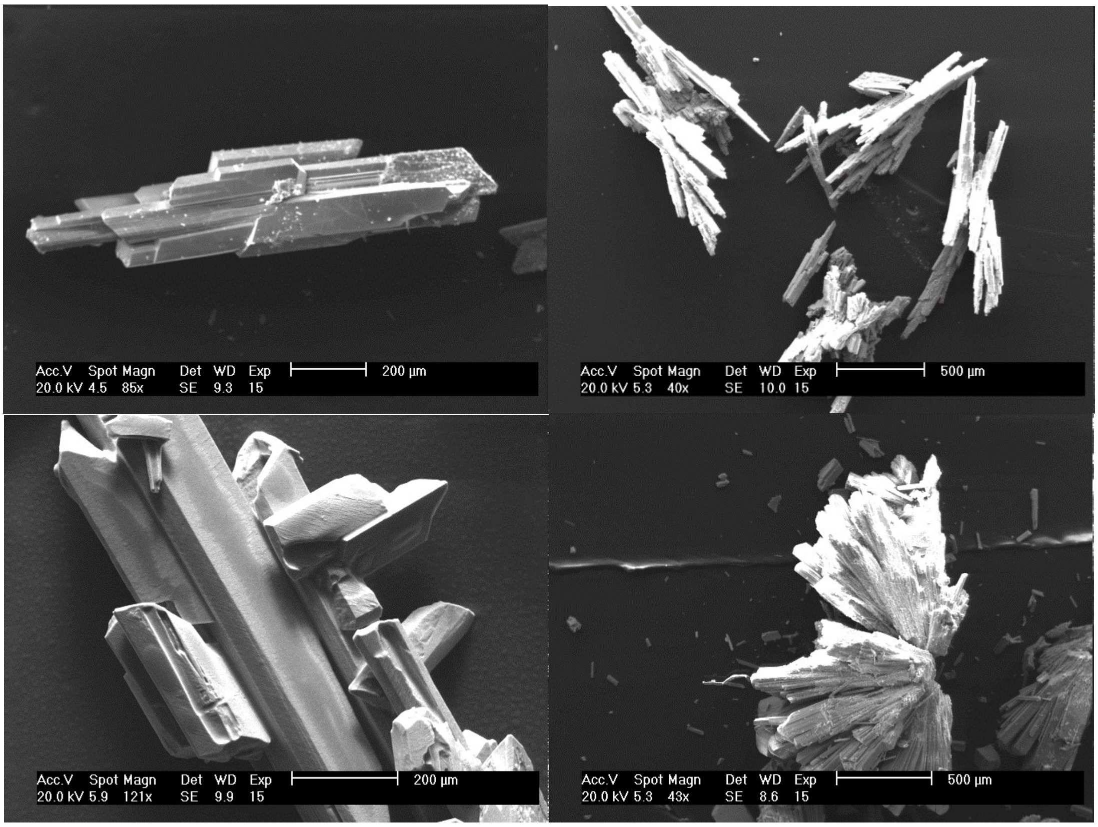

Figure S5. SEM pictures of compound **I** (top left), pseudopolymorphic compound **IV** (top right), bottom left: **II** and d) bottom right: **III**.

5. Calibration of the UV/Vis Absorption at 320 nm for Solubility Studies

The absorption vs. concentration calibration curve was obtained by dissolving **I** in different, defined concentrations in water ($1.31 \cdot 10^{-5}$ M, $2.30 \cdot 10^{-5}$ M, $3.28 \cdot 10^{-5}$ M, $4.27 \cdot 10^{-5}$ M, $5.58 \cdot 10^{-5}$ M) and measuring the UV/Vis spectra of these solutions. The peak maximum at 320 nm was evaluated and its absorption plotted against the sample concentration. A saturated solution of **I** in water was diluted by a factor of 1:25 to be in the concentration range of the calibration curve. The concentration was determined from its absorption at 320 nm and calculated back to that of the to determine the maximal solubility of **I** in water (1.19 g L^{-1}).

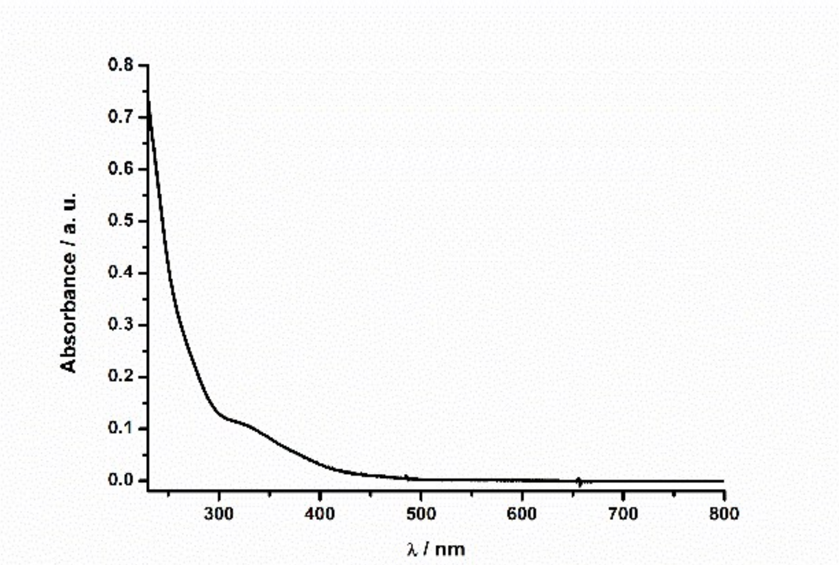

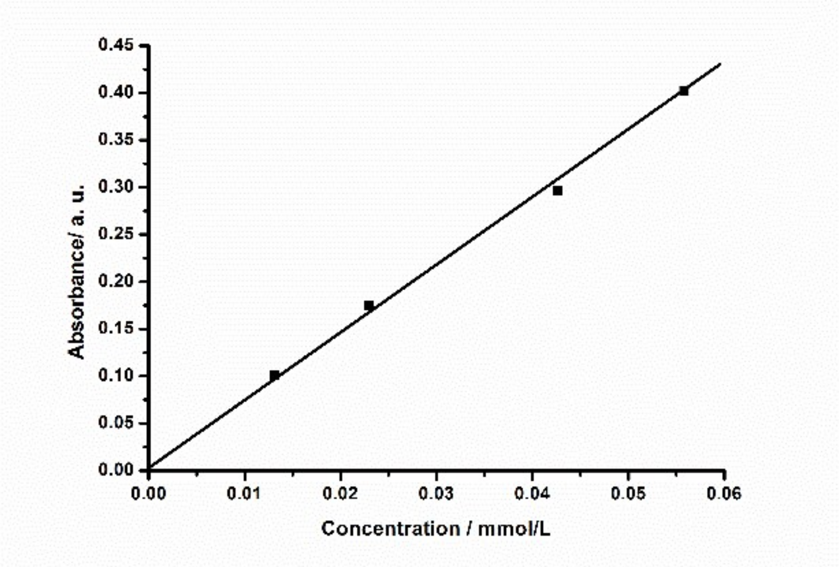

Figure S6. UV/Vis spectrum of **I** dissolved in water (top) and the calibration curve obtained from the peak maxima at 320 nm used to determine the maximal solubility (bottom).

6. Crystallographic Data

Table S2 summarizes information on single crystal structure refinement and shows clearly the similarity of the isostructural compounds **I** – **III** and the differences to the (pseudo)polymorphic compound **IV**.

Table S2 Selected crystal data and details of the structure refinement.

	**III**	**II**	**I**	**IV**
Formula	$C_{18}H_{92}N_{18}Fe_3$ $V_{15}Sb_6O_{52}$	$C_{18}H_{92}N_{18}Co_3$ $V_{15}Sb_6O_{52}$	$C_{18}H_{92}N_{18}Ni_3$ $V_{15}Sb_6O_{52}$	$C_{18}H_{74}N_{18}Ni_3$ $V_{15}Sb_6O_{43}$
MW / g·mol^{-1}	3055.24	3064.48	3063.82	2901.67
crystal system	monoclinic	monoclinic	monoclinic	trigonal
space group	*C*2	*C*2	*C*2	*P*321
a /Å	18.1991(4)	18.2302(14)	18.2404(7)	23.7437(5)
b / Å	22.6260(6)	22.6142(16)	22.7636(7)	23.7437(5)
c / Å	14.3614(3)	14.4067(11)	14.3542(5)	14.9586(3)
α / °	90	90	90	90
β / °	126.1930(10)	126.360(7)	126.449(3)	90
γ / °	90	90	90	120
V / Å^3	4772.49(19)	4783.0(6)	4794.2(3)	7303.3(3)
T / K	200	200	293	200
Z	2	2	2	3
D_{calc} / Mg·m^3	2.114	2.115	2.110	1.9767
μ / mm^{-1}	3.582	3.639	3.700	3.631
θ_{max} / °	27.94	28.00	27.00	25.00
measured refl.	37515	19592	14462	38040
unique refl.	11411	10404	8392	8614
R_{int}	0.0448	0.0647	0.0274	0.0717
refl. with $F_0>4\sigma(F_0)$	11411	10404	8392	8614
Parameters	503	503	503	463
$R_1[F_0>4\sigma(F_0)]$	0.0324	0.0453	0.0382	0.0580
wR_2 (all refl.)	0.0768	0.1187	0.0941	0.1438
GOF	1.058	1.045	1.027	0.987
$\Delta\rho_{max/min}$ /e·Å^{-3}	1.169/-0.922	1.867/-1.609	1.249/-1.070	1.338/-0.862

Flack-x-paramter	-0.02(2)	-0.07(3)	-0.006(19)	-0.028(19)

Bond valence sum (BVS) [2] yield following values:

Compound **I**: V 3.95 – 4.06, average: 4.00 and Sb 3.21 – 3.46, average: 3.37;

Compound **II**: V 3.90 – 4.06, average: 3.99 and Sb 3.21 – 3.48, average: 3.37;

Compound **III**: V 3.92 – 4.04, average 4.00 and Sb 3.38 – 3.61, average 3.51;

Compound **IV** V 3.82 – 4.12, average: 4.01 and Sb 3.29 – 3.53, average: 3.45.

All values are in the typical range of antimonato-polyoxovanadates.[1b,c,d,e,h]

The V-O bond lengths can be divided into four groups (Tables S3 – S6): Oa terminal V=O, O_b Sb-μ-O between two Sb atoms; O_c Sb/V-μ-O between two V and one Sb atom; O_d V-μ-O with only V atoms are involved.

Table S3 Bond lengths of the four different oxygen atom types of **I** in Å.

Type	Atom	V	V	V	Sb	Sb
Oa	O7	1.606(6)				
Oa	O9	1.596(8)				
Oa	O11	1.619(5)				
Oa	O13	1.620(6)				
Oa	O16	1.611(6)				
Oa	O19	1.609(5)				
Oa	O20	1.615(6)				
Oa	O21	1.600(5)				
Ob	O1				1.933(6)	1.909(6)
Ob	O22				1.926(4)	1.926(4)
Oc	O2	1.967(6)	2.009(5)		1.945(5)	
Oc	O3	1.966(5)	1.982(6)		1.977(5)	
Oc	O4	1.980(5)	1.983(5)		1.948(5)	
Oc	O5	1.975(6)	2.006(6)		1.927(5)	
Oc	O14	1.987(5)	1.984(6)		1.932(5)	
Oc	O17	2.011(6)	1.967(6)		1.953(6)	
Od	O6	1.913(5)	1.950(5)	1.930(5)		
Od	O8	1.955(5)	1.932(4)	1.955(5)		
Od	O10	1.931(6)	1.916(5)	1.927(6)		
Od	O12	1.941(6)	1.929(5)	1.957(5)		
Od	O15	1.922(5)	1.937(5)	1.946(5)		
Od	O18	1.912(6)	1.913(5)	1.940(6)		

Table S4 Bond lengths of the four different oxygen atom types of **II** in Å.

Type	Atom	V	V	V	Sb	Sb
Oa	O7	1.615(3)				
Oa	O9	1.597(7)				
Oa	O11	1.624(5)				
Oa	O13	1.628(5)				
Oa	O16	1.616(5)				
Oa	O19	1.621(5)				
Oa	O20	1.614(6)				
Oa	O21	1.600(6)				
Ob	O1				1.958(5)	1.921(6)
Ob	O22				1.939(3)	1.939(3)
Oc	O2	1.969(5)	2.007(5)		1.953(5)	
Oc	O3	1.960(5)	1.975(5)		2.007(5)	
Oc	O4	1.979(5)	1.981(5)		1.963(5)	
Oc	O5	1.969(5)	2.003(6)		1.942(6)	
Oc	O14	1.971(6)	1.986(6)		1.956(6)	
Oc	O17	2.012(6)	1.981(6)		1.953(6)	
Od	O6	1.927(5)	1.954(5)	1.920(5)		
Od	O8	1.955(5)	1.931(5)	1.963(5)		
Od	O10	1.928(5)	1.916(5)	1.940(5)		
Od	O12	1.945(6)	1.932(6)	1.956(6)		
Od	O15	1.943(5)	1.944(5)	1.926(5)		
Od	O18	1.914(5)	1.922(5)	1.939(5)		

Table S5 Bond lengths (Å) of the four different oxygen atom types of **III** in Å.

Type	Atom	V	V	V	Sb	Sb
Oa	O7	1.607(3)				
Oa	O9	1.612(5)				
Oa	O11	1.616(3)				
Oa	O13	1.628(4)				
Oa	O16	1.618(4)				
Oa	O19	1.619(4)				
Oa	O20	1.622(4)				
Oa	O21	1.598(4)				
Ob	O1				1.950(4)	1.928(4)
Ob	O22				1.942(3)	1.942(3)
Oc	O2	1.966(3)	2.007(3)		1.952(3)	
Oc	O3	1.960(3)	1.966(3)		2.005(3)	
Oc	O4	1.975(3)	1.978(3)		1.964(3)	
Oc	O5	1.967(4)	2.001(4)		1.941(3)	
Oc	O14	1.974(4)	1.972(4)		1.958(4)	
Oc	O17	2.007(4)	1.982(4)		1.950(4)	
Od	O6	1.913(3)	1.946(3)	1.929(3)		
Od	O8	1.951(3)	1.937(3)	1.960(3)		
Od	O10	1.933(4)	1.907(4)	1.929(4)		
Od	O12	1.942(4)	1.930(4)	1.959(4)		
Od	O15	1.950(4)	1.942(4)	1.916(4)		
Od	O18	1.914(4)	1.918(4)	1.938(4)		

Table S6 Bond lengths of the four different oxygen atom types in Å of **IV**.

Type	Atom	V	V	V	Sb	Sb
Oa	O16	1.608(12)				
Oa	O17	1.602(12)				
Oa	O20	1.605(12)				
Oa	O21	1.628(12)				
Oa	O22	1.653(11)				
Ob	O11				1.909(14)	1.966(15)
Oc	O12	1.981(13)	2.013(14)		1.942(13)	
Oc	O13	1.943(13)	1.993(12)		1.967(13)	
Oc	O14	1.966(12)	1.956(13)		1.984(12)	
Oc	O15	1.994(13)	2.003(14)		1.938(14)	
Od	O18	1.896(12)	1.920(12)	1.933(13)		
Od	O19	1.917(11)	1.927(11)	1.952(10)		
Od	O23	1.872(12)	1.915(12)	1.956(12)		
Od	O24	1.888(12)	1.958(12)	1.965(12)		

Figure S7 shows the arrangement of the (pseudo)polymorph compound **IV** with its discrete cluster anions and the Ni amine complexes as counter cations.

Table S7 shows the TM-N bond length and angles. All values are in typical ranges for known TM amine complex acting as counter cations in heteroatom incorporated POVs.

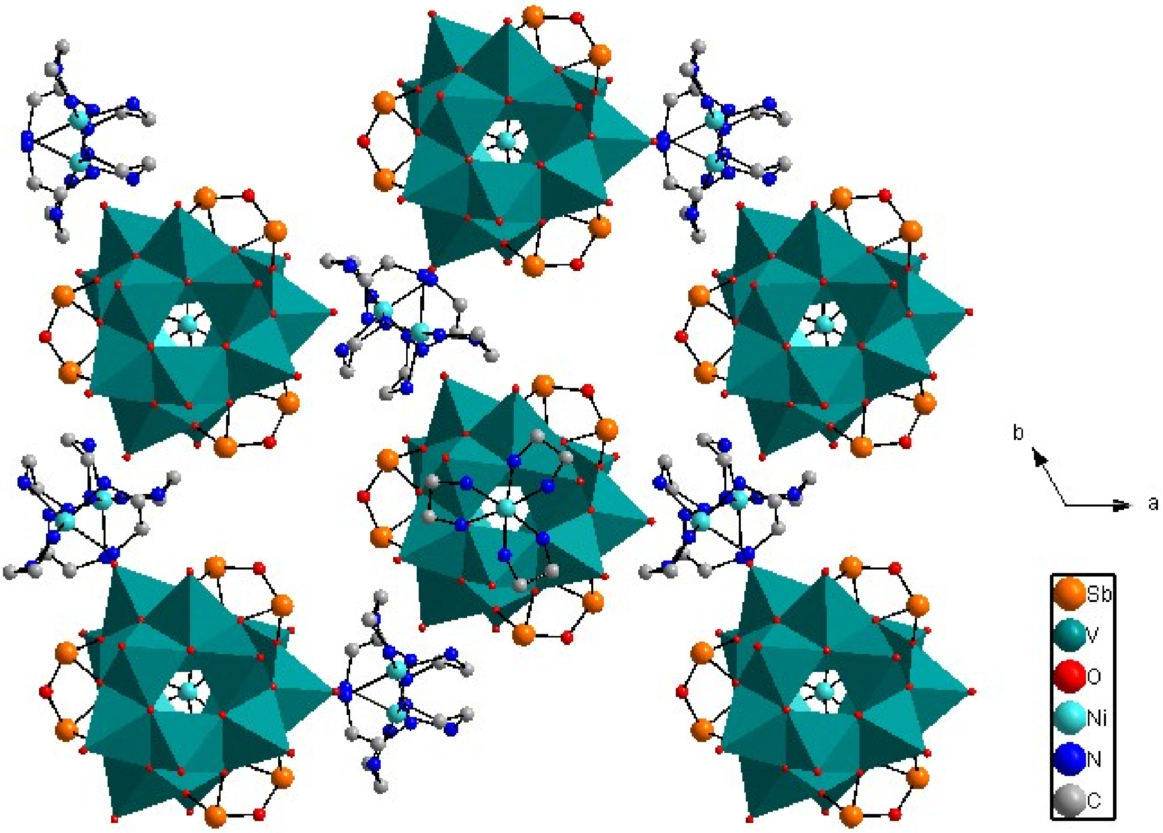

Figure S7. Arrangement of the cluster anions and transition metal complex cations of **IV**. Hydrogen atoms are not displayed for clarity.

Table S7 Selected bond lengths and bond angles of the $Ni(en)_3^{2+}$ cations in the crystal structure of compound **I**.

**Bond Lengths**		**Bond Angles**	
Ni(1)-N(2)	2.122(6)	N(2)-Ni(1)-N(11)	92.0(3)
Ni(1)-N(11)	2.124(9)	N(2)-Ni(1)-N(22)	92.1(3)
Ni(1)-N(22)	2.134(9)	N(11)-Ni(1)-N(22)	92.1(3)
Ni(1)-N(1)	2.139(8)	N(2)-Ni(1)-N(1)	80.1(3)
Ni(1)-N(12)	2.140(7)	N(11)-Ni(1)-N(1)	96.7(3)
Ni(1)-N(21)	2.146(8)	N(22)-Ni(1)-N(1)	168.4(3)
Ni(2)-N(41)	2.119(8)	N(2)-Ni(1)-N(12)	170.1(3)
Ni(2)-N(32)	2.123(9)	N(11)-Ni(1)-N(12)	81.3(3)
Ni(2)-N(31)	2.131(8)	N(41)-Ni(2)-N(32)	171.5(4)
		N(41)-Ni(2)-N(31)	93.4(4)
		N(32)-Ni(2)-N(31)	81.1(4)
		N(22)-Ni(1)-N(12)	95.3(3)
		N(1)-Ni(1)-N(12)	93.5(3)
		N(2)-Ni(1)-N(21)	94.4(3)
		N(11)-Ni(1)-N(21)	170.5(3)
		N(22)-Ni(1)-N(21)	80.6(3)
		N(1)-Ni(1)-N(21)	91.3(3)
		N(12)-Ni(1)-N(21)	93.2(3)

Table S8 Selected bond lengths and angles of the $Co(en)_3^{2+}$ cations in the crystal structure of compound **II**.

**Bond Lengths**		**Bond Angles**	
Co(1)-N(1)	2.201(7)	N(11)-Co(1)-N(1)	97.7(3)
Co(1)-N(2)	2.162(7)	N(12)-Co(1)-N(1)	92.4(3)
Co(1)-N(11)	2.169(7)	N(22)-Co(1)-N(1)	166.9(3)
Co(1)-N(12)	2.172(7)	N(21)-Co(1)-N(1)	90.9(3)
Co(1)-N(22)	2.184(7)	N(32)-Co(2)-N(41)	171.4(3)
Co(1)-N(21)	2.186(8)	N(32)-Co(2)-N(31)	80.1(3)
Co(2)-N(32)	2.168(8)	N(41)-Co(2)-N(31)	93.6(3)
Co(2)-N(31)	2.180(8)	N(2)-Co(1)-N(11)	91.5(3)
		N(2)-Co(1)-N(12)	168.4(3)
		N(11)-Co(1)-N(12)	80.7(3)
		N(2)-Co(1)-N(22)	91.6(3)
		N(11)-Co(1)-N(22)	92.6(3)
		N(12)-Co(1)-N(22)	97.3(3)
		N(2)-Co(1)-N(21)	95.3(3)
		N(11)-Co(1)-N(21)	169.8(3)
		N(12)-Co(1)-N(21)	93.6(3)
		N(22)-Co(1)-N(21)	79.7(3)
		N(2)-Co(1)-N(1)	80.1(3)

Table S9 Selected bond lengths and angles of the $Fe(en)_3^{2+}$ cations in the crystal structure of compound **III**.

Bond Lengths		Bond Angles	
Fe(1)-N(2)	2.193(5)	N(2)-Fe(1)-N(21)	96.74(18)
Fe(1)-N(12)	2.210(5)	N(12)-Fe(1)-N(21)	93.5(2)
Fe(1)-N(11)	2.211(5)	N(11)-Fe(1)-N(21)	167.40(19)
Fe(1)-N(22)	2.223(5)	N(22)-Fe(1)-N(21)	78.23(18)
Fe(1)-N(21)	2.234(5)	N(31)-Fe(2)-N(32)	78.4(2)
Fe(1)-N(1)	2.237(5)	N(31)-Fe(2)-N(41)	94.1(2)
Fe(2)-N(31)	2.208(5)	N(32)-Fe(2)-N(41)	169.6(2)
Fe(2)-N(32)	2.210(5)	N(2)-Fe(1)-N(12)	166.43(19)
Fe(2)-N(41)	2.215(5)	N(2)-Fe(1)-N(11)	91.9(2)
		N(12)-Fe(1)-N(11)	79.6(2)
		N(2)-Fe(1)-N(22)	91.29(19)
		N(12)-Fe(1)-N(22)	99.5(2)
		N(11)-Fe(1)-N(22)	92.5(2)
		N(2)-Fe(1)-N(1)	78.44(18)
		N(12)-Fe(1)-N(1)	92.4(2)
		N(11)-Fe(1)-N(1)	98.9(2)
		N(22)-Fe(1)-N(1)	164.87(19)
		N(21)-Fe(1)-N(1)	91.8(2)

Table S10 Selected bond lengths and angles of the $Ni(en)_3^{2+}$ cations in the crystal structure of compound **IV**.

**Bond Lengths**		**Bond Angles**	
Ni(1)-N(1)#7	2.110(17)	N(9)-Ni(3)-N(9)#4	92.6(12)
Ni(1)-N(1)#8	2.110(17)	N(9)-Ni(3)-N(9)#9	79(2)
Ni(1)-N(1)	2.110(17)	N(9)#4-Ni(3)-N(9)#9	167(3)
Ni(1)-N(2)#7	2.119(17)	N(9)-Ni(3)-N(9)#10	167(3)
Ni(1)-N(2)	2.119(17)	N(9)#4-Ni(3)-N(9)#10	98(2)
Ni(1)-N(2)#8	2.119(17)	N(9)#9-Ni(3)-N(9)#10	92.6(12)
Ni(11)-N(3)	2.110(13)	N(9)-Ni(3)-N(9)#11	98(2)
Ni(11)-N(6)	2.112(19)	N(9)#4-Ni(3)-N(9)#11	79(2)
Ni(11)-N(8)	2.12(2)	N(9)#9-Ni(3)-N(9)#11	92.6(12)
Ni(11)-N(5)	2.121(16)	N(9)#10-Ni(3)-N(9)#11	92.6(12)
Ni(11)-N(4)	2.12(2)	N(9)-Ni(3)-N(9)#3	92.6(12)
Ni(11)-N(7)	2.14(2)	N(9)#4-Ni(3)-N(9)#3	92.6(12)
Ni(3)-N(9)#4	2.10(3)	N(9)#9-Ni(3)-N(9)#3	98(2)
Ni(3)-N(9)#9	2.10(3)	N(9)#10-Ni(3)-N(9)#3	79(2)
Ni(3)-N(9)#10	2.10(3)	N(1)#7-Ni(1)-N(1)#8	92.0(6)
Ni(3)-N(9)#11	2.10(3)	N(1)#7-Ni(1)-N(1)	92.0(6)
Ni(3)-N(9)#3	2.10(3)	N(1)#8-Ni(1)-N(1)	92.0(6)
		N(1)#7-Ni(1)-N(2)#7	173.6(7)
		N(1)#8-Ni(1)-N(2)#7	91.7(7)
		N(1)-Ni(1)-N(2)#7	82.7(6)
		N(1)#7-Ni(1)-N(2)	91.7(7)
		N(1)#8-Ni(1)-N(2)	82.7(6)
		N(1)-Ni(1)-N(2)	173.6(7)
		N(2)#7-Ni(1)-N(2)	94.0(6)
		N(1)#7-Ni(1)-N(2)#8	82.7(6)
		N(1)#8-Ni(1)-N(2)#8	173.6(7)
		N(1)-Ni(1)-N(2)#8	91.7(7)
		N(2)#7-Ni(1)-N(2)#8	94.0(6)
		N(2)-Ni(1)-N(2)#8	94.0(6)
		N(3)-Ni(11)-N(6)	170.5(7)
		N(3)-Ni(11)-N(8)	93.6(7)

Table S10 continued

	N(6)-Ni(11)-N(8)	94.8(8)
	N(3)-Ni(11)-N(5)	91.4(6)
	N(6)-Ni(11)-N(5)	81.0(8)
	N(8)-Ni(11)-N(5)	170.6(7)
	N(3)-Ni(11)-N(4)	82.7(7)
	N(6)-Ni(11)-N(4)	92.6(8)
	N(8)-Ni(11)-N(4)	91.4(7)
	N(5)-Ni(11)-N(4)	97.2(8)
	N(3)-Ni(11)-N(7)	90.2(8)
	N(6)-Ni(11)-N(7)	95.6(8)
	N(8)-Ni(11)-N(7)	80.0(8)
	N(5)-Ni(11)-N(7)	92.0(8)
	N(4)-Ni(11)-N(7)	168.6(7)
	N(9)#11-Ni(3)-N(9)#3	167(3)

The discrete cluster anions and the discrete $M(en)_3^{2+}$ cations form a complex hydrogen network. The hydrogen bond lengths and the corresponding interacting atoms for all four compounds are listed in Tables S8 – S11.

Table S11 Hydrogen bonds with H-A < r(A) + 2.000 Å and °DHA > 110 ° of compound **I**.

D-H	d(D-H)	d(H-A)	°DHA	d(D-A)	A
N1-H1N	0.920	2.357	141.34	3.128	O21 [x, y, z+1]
N2-H3N	0.920	2.022	164.17	2.918	O16
N2-H4N	0.920	2.283	167.58	3.188	O32 [-x+2, y, -z+2]
N11-H5N	0.920	2.206	154.72	3.063	O20 [x, y, z+1]
N11-H6N	0.920	2.212	153.73	3.064	O7 [-x+3/2, y+1/2, -z+1]
N12-H7N	0.920	2.093	155.43	2.954	O9 [x-1/2, y+1/2, z]
N12-H8N	0.920	2.172	152.56	3.019	O31 [-x+1, y, -z+1]
N21-H9N	0.920	2.535	140.58	3.298	O16
N22-H11N	0.920	2.361	150.53	3.194	O8 [-x+3/2, y+1/2, -z+1]
N31-H13N	0.920	2.188	163.43	3.081	O11 [-x+1, y, -z]
N31-H14N	0.920	2.360	148.09	3.179	O31 [-x+1, y, -z]
N32-H15N	0.920	2.245	158.72	3.121	O6
N32-H16N	0.920	2.566	118.50	3.112	O11
N41-H17N	0.920	2.425	130.19	3.099	O18 [-x+1, y, -z]
N41-H17N	0.920	2.492	148.45	3.311	O19 [-x+1, y, -z]
N41-H18N	0.920	2.220	149.86	3.050	O21 [-x+1, y, -z]

TableS12. Hydrogen bonds with H-A < r(A) + 2.000 Å and °DHA > 110 ° of compound **II**.

D-H	d(D-H)	d(H-A)	°DHA	d(D-A)	A
N1-H1N	0.920	2.353	139.09	3.107	O21 [x, y, z+1]
N2-H3N	0.920	2.021	165.98	2.922	O16
N2-H4N	0.920	2.302	164.36	3.197	O32 [-x+2, y, -z+2]
N11-H5N	0.920	2.202	152.22	3.046	O20 [x, y, z+1]
N11-H6N	0.920	2.245	152.02	3.088	O7 [-x+3/2, y+1/2, -z+1]
N12-H7N	0.920	2.098	156.55	2.964	O9 [x-1/2, y+1/2, z]
N12-H8N	0.920	2.248	150.48	3.082	O31 [-x+1, y, -z+1]
N21-H9N	0.920	2.482	142.36	3.259	O16
N22-H11N	0.920	2.389	147.98	3.206	O8 [-x+3/2, y+1/2, -z+1]
N31-H13N	0.920	2.196	161.51	3.083	O11 [-x+1, y, -z]
N31-H14N	0.920	2.362	147.25	3.175	O31 [-x+1, y, -z]
N32-H15N	0.920	2.242	160.50	3.124	O6
N32-H16N	0.920	2.538	118.68	3.087	O11
N41-H17N	0.920	2.429	130.14	3.103	O18 [-x+1, y, -z]
N41-H17N	0.920	2.492	148.81	3.314	O19 [-x+1, y, -z]
N41-H18N	0.920	2.214	150.98	3.051	O21 [-x+1, y, -z]

Table S13. Hydrogen bonds with H-A < r(A) + 2.000 Å and °DHA > 110 ° of compound **III**.

D-H	d(D-H)	d(H..A)	°DHA	d(D..A)	A
N1-H2N	0.900	2.383	138.19	3.112	O21 [x, y, z-1]
N2-H3N	0.900	2.386	164.69	3.262	O32 [-x, y, -z]
N2-H4N	0.900	2.040	165.97	2.921	O16
N11-H5N	0.900	2.313	150.79	3.130	O7 [-x+1/2, y-1/2, -z+1]
N11-H6N	0.900	2.219	152.64	3.046	O20 [x, y, z-1]
N12-H7N	0.900	2.274	151.69	3.096	O31 [-x+1, y, -z+1]
N12-H8N	0.900	2.109	154.88	2.949	O9 [x+1/2, y-1/2, z]
N21-H10N	0.900	2.489	143.94	3.260	O16
N22-H12N	0.900	2.458	144.75	3.235	O8 [-x+1/2, y-1/2, -z+1]
N31-H13N	0.900	2.442	143.57	3.211	O31 [-x+1, y, -z+2]
N31-H14N	0.900	2.246	161.54	3.113	O11 [-x+1, y, -z+2]
N32-H15N	0.900	2.539	122.39	3.115	O11
N32-H16N	0.900	2.276	156.25	3.121	O6
N41-H17N	0.900	2.248	149.94	3.060	O21 [-x+1, y, -z+2]
N41-H18N	0.900	2.477	132.08	3.151	O18 [-x+1, y, -z+2]
N41-H18N	0.900	2.546	148.99	3.349	O19 [-x+1, y, -z+2]

Table S14. Hydrogen bonds with H..A < r(A) + 2.000 Å and <DHA > 110 ° of compound **IV**.

D-H	d(D-H)	d(H..A)	°DHA	d(D..A)	A
N1-H1N1	0.990	2.359	146.28	3.230	O22 [y, x, -z+2]
N1-H1N1	0.990	2.316	135.82	3.104	O23 [x-y+1, -y+1, -z+2]
N1-H2N1	0.990	2.307	142.40	3.150	O22 [x-y+1, -y+1, -z+2]
N2-H1N2	0.990	2.334	144.61	3.192	O17 [-x+1, -x+y, -z+1]
N2-H1N2	0.990	2.321	138.51	3.132	O18 [x-y+1, -y+1, -z+1]
N2-H2N2	0.990	2.397	133.80	3.164	O17 [x-y+1, -y+1, -z+1]
N3-H1N3	0.990	1.996	164.95	2.963	O20 [-x+y, -x+1, z]
N4-H1N4	0.990	2.401	133.90	3.169	O17 [-x+1, -x+y, -z+1]
N5-H1N5	0.990	2.183	149.08	3.075	O16 [y, x, -z+1]
N5-H2N5	0.990	2.297	148.78	3.185	O4 [y, x, -z+2]
N6-H1N6	0.990	1.972	154.28	2.895	O5
N7-H1N7	0.990	2.414	147.12	3.290	O6
N8-H1N8	0.990	2.378	145.14	3.240	O20 [-x+y, -x+1, z]
N9-H1N9	0.990	2.195	159.46	3.141	O7
N9-H1N9	0.990	2.477	123.67	3.136	O8
N9-H2N9	0.990	2.414	149.25	3.304	O7 [-x+y+1, -x+2, z]

7. Magnetic Properties

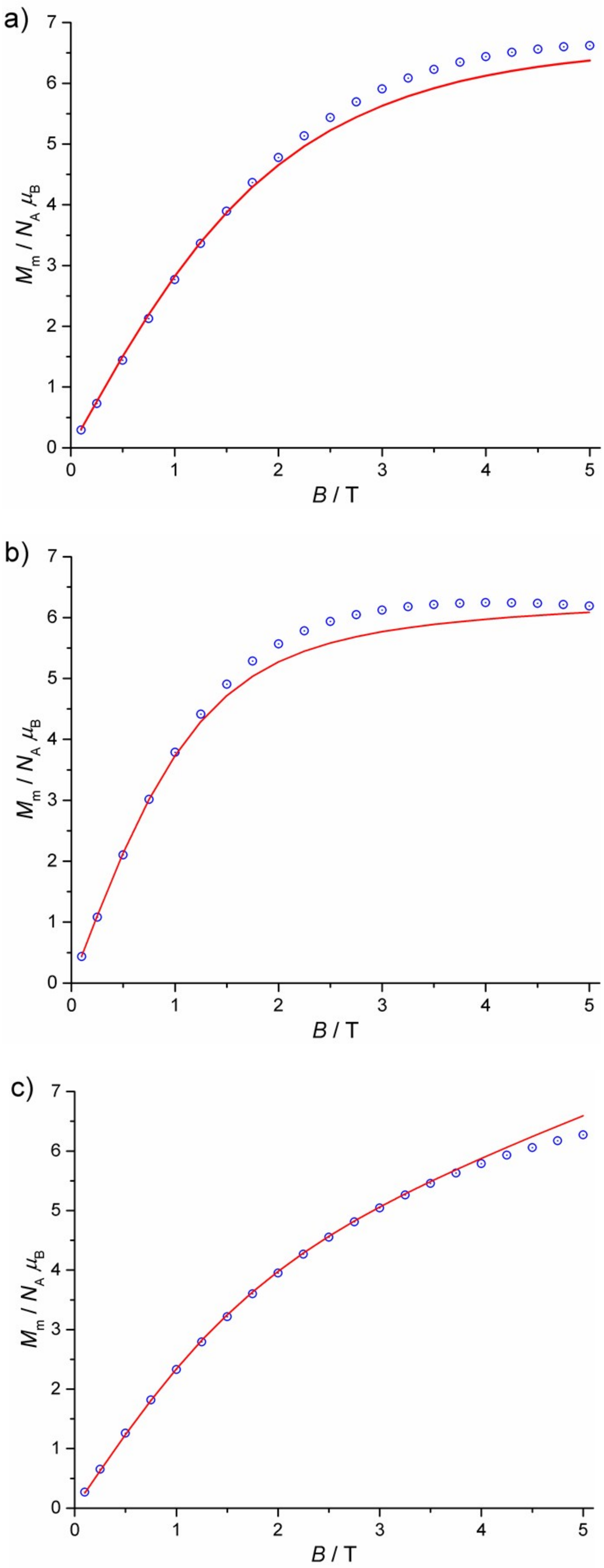

Figure S8. Molar magnetization M_m as a function of the applied field B: $M_m(exp) - M_m([V_{15}Sb_6O_{42}]^{6-})$ for compound **I** (a), **II** (b), **III**), shown as blue open circles;
best fits: red lines.

Table S15. Parameters of the "full model" simulations of **I** – **III**.

**Parameter**	**I** (M = Ni)	**II** (M = Co)	**III** (M = Fe)
Racah *B* [a] / cm^{-1}	1084	1115	1058
Racah *C* [a] / cm^{-1}	4831	4366	3901
ζ_{3d} [a] / cm^{-1}	649	533	410
*B***Error!** [b] / cm^{-1}	–24307 ± 484	–842 ± 80	13426 ± 43
*B***Error!** [b] / cm^{-1}	26213 ± 98	43382 ± 50	39262 ± 15
*B***Error!** [b] / cm^{-1}	13898 ± 167	25154 ± 16	10064 ± 35
zJ' [c] / cm^{-1}	–0.01 ± 0.01	+0.01 ± 0.01	–0.53 ± 0.01
SQ [d]	2.1%	2.6%	1.6%

[a] Griffith, J.S. *The Theory of Transition-Metal Ions*, Cambridge University Press, Cambridge, 1971; [b] ligand field parameter in Wybourne notation; [c] mean field parameter ("–2*J*" notation) [d] goodness of fit.

8. Electrospray ionization mass spectrometry

Besides the known signals of [**M**]$^{3-}$/[**M**·H]$^{3-}$, [**M**·H_2O]$^{3-}$/[**M**·H·H_2O]$^{3-}$, [**M**·Ni(en)]$^{2-}$/ [**M**·H·Ni(en)]$^{2-}$, [**M**·Ni(en)·H_2O]$^{2-}$/[**M**·H·Ni(en)·H_2O]$^{2-}$ and [**N**·H_2O]$^{3-}$/[**N**·H·H_2O]$^{3-}$ at *m/z* 722, 728, 1142, 1151 and 792, respectively, in the ESI-Q-TOF-HRMS spectra of {$Ni(en)_3$}$_3$[$V_{15}Sb_6O_{42}$], a series of peaks exists for which no conclusive assignment could be made (*m/z* 671, 677, 696, 702, 766, 1104, 1113).

In all experiments performed (ESI mass spectra at different ionization conditions, H/D- and ^{16}O/^{18}O-exchange experiments, collision-induced fragmentation experiments), these ions behave in close analogy to the parent cluster. It can thus be assumed that they belong to structurally closely related cluster species, for which we were nevertheless unable to find a fully convincing elemental composition, which is in line with all experimental data.

As the powder diffraction patterns of a sample that was used for the mass spectrometric experiments clearly showed the sample not to contain significant amounts of impurities, we assume that these signals correspond to a marginal level of impurities, which are more easily ionized and thus appear with higher intensities in the mass spectra than expected from their abundance in the sample.

9. References

(1) (a) Y. Gao, Z. Han, Y. Xu, C. Hu, *J. Clust. Sci.*, 2010, **21**, 163; (b) E. Antonova C. Näther, P. Kögerler, W. Bensch, *Angew. Chem.*, 2011, **123**, 790 ; (c) R. Kiebach, C. Näther, P. Kögerler, W. Bensch, *Dalton Trans.,* 2007, 3221; (d) E. Antonova, C. Näther, W. Bensch, *Dalton Trans.,* 2012, **41**, 1338; (e) E. Antonova, C. Näther, W. Bensch, *CrystEngComm.,* 2012, **14**, 6853; (f) E. Antonova, C. Näther, P. Kögerler, W. Bensch, *Dalton Trans*., 2012, **41**, 6957; (g) L. Yu, J.-P. Liu, J.-P. Wang, J.-Y. Niu, *Chem. Res. Chinese Universities,* 2009, **25**, 426; (h) E. Antonova, C. Näther, P. Kögerler, W. Bensch, *Inorg. Chem.,* 2012, **51**, 2311.

(2) M. O'Keeffe, N. E. Brese, *J. Am. Chem. Soc*., 1991, **113**, 3226.
